# Towards Autonomous Bio-Inspired Optimization: Deep Reinforcement Learning for Adaptive Metaheuristic Orchestration

**DOI:** 10.3390/biomimetics11090682

**Published:** 2026-09-21

**Authors:** César Carrasco, Broderick Crawford, Carlos Valle, Roberto Zulantay, Giovanni Giachetti, Ricardo Soto

**Affiliations:** 1 Escuela de Ingeniería Informática, Pontificia Universidad Católica de Valparaíso, Avenida Brasil 2241, Valparaíso 2362807, Chile; carlos.valle@pucv.cl (C.V.); roberto.zulantay.a@mail.pucv.cl (R.Z.); ricardo.soto@pucv.cl (R.S.); 2 Facultad de Ingeniería, Universidad Andrés Bello, Antonio Varas 880, Providencia, Santiago 7591538, Chile; giovanni.giachetti@unab.cl

**Keywords:** nature-inspired optimization, bio-inspired metaheuristics, swarm intelligence, evolutionary computation, hyper-heuristics, deep reinforcement learning, metaheuristic selection, multidimensional knapsack problem, set covering problem

## Abstract

Learning-based hyper-heuristics for combinatorial optimization select from among low-level operators or tune the parameters of a single metaheuristic. However, online selection among complete population-based metaheuristics that share one evolving population and its joint configuration through a common action space have not been formulated or evaluated for binary combinatorial optimization problems. This work presents such a formulation and evaluates it on two binary domains: the multidimensional knapsack problem (MKP), and the set covering problem (SCP). A proximal policy optimization (PPO) agent orchestrates a portfolio of seven nature-inspired population-based metaheuristics, transferring the full population between methods at each decision, then acts at every fixed quantum of iterations on a fourteen-dimensional size-independent description of the search state. Over the same state, reward, and portfolio, three action spaces of increasing richness are instantiated: discrete selection, joint selection and configuration through algorithm-specific translation maps, and heterogeneous co-evolution through a simplex allocation. The three techniques are evaluated under five-fold cross-validation with twenty recorded seeds on the Chu and Beasley cb9 set (n=500 items, B=25,000 evaluations, quantum Q=25) against the best-known values of the recent literature, and the selection technique is also evaluated on 65 set-covering instances. On cb9, the selection agent is ahead of the blind round-robin and random baselines. The co-evolution agent places second, with a 0.043 percentage point gap to the best-known value behind the strongest single metaheuristic. At half the evaluation budget, the two agents rank first and second; on the Set Covering Problem, where no single method dominates, the selection agent ranks first among eleven strategies. An untrained control and a portfolio ablation show that the policy learns mainly which members to avoid; the joint selection-and-configuration agent does not yet improve on the blind baselines. The formulation, translation maps, simplex allocation, and evaluation protocol are released with this study.

## 1. Introduction

### 1.1. Motivation

Nature-inspired population-based metaheuristics are a standard tool for large binary optimization problems. By emulating natural processes such as biological evolution, the collective behavior of swarms, and animal echolocation, they offer a diverse repertoire of search mechanisms. Their effectiveness depends on two choices that remain manual: which metaheuristic to run, and how to configure it. Both choices are instance-dependent and phase-dependent, and the no-free-lunch theorems [1] formalize why no fixed answer exists. Hyper-heuristics address this dependency by searching in the space of heuristics rather than in the space of solutions [2,3]: a high-level strategy decides during the run which low-level component should act next.

Three observations motivate this study. First, in the experiments reported below, the strongest single metaheuristic is Island GA on the cb9 instances and SHADE on the cb7 instances; as no single method leads every fold of the Set Covering Problem, a method fixed in advance will be the wrong choice on some benchmarks. Second, the population-based methods that dominate these problems are complete search procedures rather than interchangeable operators, so a selector that switches among them must hand over the population state; hyper-heuristics that select moves or operators do not need to do this. Third, an agent that makes the choice online must learn from search-state signals that are common to all methods and to both domains, which makes a single formulation possible and is what this study tests.

Seen through a biomimetic lens, each portfolio member is a specialized search agent that embodies a distinct adaptive behavior observed in nature: the selective pressure of evolution, the social coordination of a swarm, or the echolocation of dolphins. Orchestrating them is then not only an algorithm-selection problem but a question of how heterogeneous biologically-inspired behaviors can be made to cooperate. Rather than fixing that cooperation by hand, we let a learning agent discover it so that the division of labor among strategies emerges from the search itself. Under this reading, the framework is a step toward autonomous collective bio-inspired optimization in which diverse natural search behaviors are coordinated by a learned decision mechanism instead of a static rule.

### 1.2. Approach and Contributions

This article studies a selection hyper-heuristic with three design decisions that, in combination, distinguish it from prior work. First, the low-level components are seven complete nature-inspired, population-based metaheuristics spanning three biological metaphors: natural evolution with a genetic algorithm (GA), a memetic genetic algorithm (MGA) [4], an island genetic algorithm (Island GA) [5], an estimation-of-distribution algorithm (EDA) [6], and success history-based adaptive differential evolution (SHADE) [7]; swarm intelligence with particle swarm optimization (PSO) [8]; and biological echolocation with dolphin echolocation (Dolphin) [9]. Second, the full population is transferred from the active metaheuristic to the next one at every decision point, so the portfolio operates on a single evolving population. Third, the decision maker is a deep reinforcement learning (DRL) agent whose action takes three forms of increasing richness: the selection of the next metaheuristic; that selection joined to a configuration vector $\kappa \in {\left[0,1\right]}^{3}$ that algorithm-specific translation maps convert into native parameters; and a simplex allocation that co-evolves several metaheuristics in parallel. The agent observes a fourteen-dimensional size-independent description of the search state, and is trained with proximal policy optimization (PPO) [10].

The experimental study addresses two research questions (RQs). RQ1: Does the learned policy outperform the individual portfolio members and non-adaptive selection strategies on the Chu and Beasley cb9 benchmark of the multidimensional knapsack problem (MKP; n=500 items) under a reduced budget (B=25,000 evaluations, quantum Q=25)? RQ2: Does the same decision problem, with the state, reward, and portfolio unchanged, transfer to a second binary domain, namely, the Set Covering Problem (SCP), and does the answer to RQ1 depend on the domain?

A diagnostic study on the smaller cb7 set showed why RQ1 must be posed under a reduced budget: at the budget that the literature conventionally grants, the benchmark saturates, the whole portfolio reaches near-optimal solutions that no selector, learned or blind, can separate. This negative finding shapes the present cb9 design. Under this regime and under a five-fold cross-validation protocol with recorded seeds, the comparative evaluation answers RQ1 in part: the learned policy is ahead of the non-adaptive baselines but behind the two strongest portfolio members (Island GA and MGA) on cb9, whereas on the Set Covering Problem, where no member dominates, it ranks first (RQ2). The contributions are: (i) the technique itself, a shared formulation with full population transfer instantiated as discrete selection, joint selection and configuration with explicit translation maps for seven metaheuristics, and simplex co-evolution (Section 3 and Appendix A); (ii) a verified experimental infrastructure for both benchmarks, with the cb9 reference values harmonized to the best-known results of the recent literature [11,12] and an evaluation protocol with cross-validation, recorded seeds and control strategies (Section 4); and (iii) the empirical evaluation of the technique on two domains, including the regime verification that should precede training and the controls that locate the contribution of learning (Section 5 and Section 6).

## 2. Related Work: Hyper-Heuristics and Learned Metaheuristic Selection

### 2.1. Hyper-Heuristics

Burke et al. [2] classified hyper-heuristics by the nature of the search space (selection versus generation of heuristics) and the source of feedback. Selection hyper-heuristics combine a selection rule with an acceptance criterion; the choice function of Cowling et al. [13] is the canonical early example, and the survey by Drake et al. [3] organizes two decades of subsequent mechanisms, reporting that simple selectors are hard to beat and are mandatory baselines for learned approaches. Most of this literature selects among single-move or perturbation operators. Treating complete population-based metaheuristics as the unit of selection changes the problem: each component carries internal state, and the orchestrator must decide what survives a switch. Here, the population survives and the internal state is rebuilt.

### 2.2. Adaptive Operator Selection

Adaptive operator selection allocates trials among operators inside a single evolutionary algorithm. Adaptive pursuit [14] moves selection probabilities toward the operator with the best recent credit; Fialho et al. [15] analyzed bandit-based allocation and credit assignment schemes. These mechanisms are stateless beyond credit statistics and define the natural ablation point for state-conditioned policies; their inclusion as experimental baselines is left to the continuation outlined in Section 7.

### 2.3. Reinforcement Learning for Heuristic Selection and Dynamic Configuration

Kallestad et al. [16] proposed a deep reinforcement learning hyper-heuristic that selects among low-level heuristics across several combinatorial domains. Closer to our setting, Guo et al. [17] selected dynamically among differential evolution variants with population inheritance and search-state features, demonstrating deep reinforcement learning over complete optimizers on continuous problems. The dynamic algorithm configuration framework [18,19] formalizes online parameter control as a contextual Markov decision process (MDP) and sets a methodological requirement that any configuration claim must eventually meet: comparison against the best static configuration, not only against defaults. Reward design is a known difficulty: shaping terms accelerate learning but can displace the objective, and the policy-invariance results of Ng et al. [20] characterize which shaping functions are safe. Seed sensitivity and reporting standards for deep reinforcement learning are documented by Henderson et al. [21]. More recent work moves learning to the design of the algorithm itself: Yi et al. [22] trained a reinforcement learning agent to compose the algorithmic components of a general search framework, and the survey of Ma et al. [23] organizes this line of meta-black-box optimization. Karimi-Mamaghan et al. [24] also reviewed how machine learning has been placed at the service of metaheuristics for combinatorial problems. For the binary combinatorial problems addressed here, learning has been applied inside a single metaheuristic: to the choice of the binarization scheme through a reinforcement learning policy [25], to the autonomous balance of the parameters of population-based methods through a self-adaptive learned strategy [26], and to the transfer function that binarizes continuous methods [27]. These works learn over moves, over the parameters or components of one algorithm, or over variants of one algorithm family on continuous problems; none learns to select and configure complete heterogeneous population-based metaheuristics over a single transferred population.

### 2.4. Multi-Method Population Search

AMALGAM (a multialgorithm genetically adaptive method) [28] runs several metaheuristics concurrently on one population, each contributing offspring in proportion to recent survival; population-based algorithm portfolios [29] divide the budget among constituent algorithms with migration; the meta-hyper-heuristics of Grobler et al. [30] select among complete metaheuristics over a common population with adaptive selectors. These methods allocate by fixed adaptation rules driven by recent success. The combination studied here, offline-trained state-conditioned orchestration with full population transfer, in its selection, selection-and-configuration and simplex-allocation forms, has to our knowledge not been examined on the multidimensional knapsack or the set covering problem; the selection-only and allocation-rule components have the antecedents above.

### 2.5. The Benchmark Problems

Puchinger et al. [31] reviewed the structure and algorithms of the 0–1 multidimensional knapsack problem. The benchmark of Chu and Beasley [32], distributed through OR-Library [33], remains the standard; the strongest published results on the large sets belong to the two-phase tabu-evolutionary algorithm of Lai et al. [11], with earlier records by Vasquez and Vimont [34] and Boussier et al. [35]. Drake et al. [36], evaluated selection hyper-heuristics on this same benchmark, providing the closest experimental anchor for our setting.

Research on the MKP has remained active since. Lai et al. [37] proposed a diversity-preserving quantum particle swarm optimizer evaluated on the full 270-instance Chu–Beasley benchmark; Jovanovic and Voß [38] combined fixed set search with integer programming, approaching the solution quality of the two-phase tabu-evolutionary algorithm at roughly one fifth of its computational cost; and Xu et al. [12] hybridized evolutionary search with exact exploration of promising subspaces, improving the best-known value of three of the thirty 30×500 instances, the only such improvements published since 2018. Cacchiani et al. [39] survey the wider body of multidimensional knapsack research. These methods are single algorithms engineered for the MKP; none addresses the online algorithm selection problem studied here, which is orthogonal to them and can incorporate any of them as portfolio members.

The Set Covering Problem, the second domain of this study, has a benchmark of the same origin: the OR-Library sets of Beasley [40,41], with proven optima for most sets. Recent work on these instances includes the local-branching heuristic of Beasley [42] and, in this journal, bio-inspired binary methods evaluated on subsets of the same battery: a binary growth optimizer [43], a binary dream optimization algorithm with data-driven repair [44] and chaotic maps that regulate exploration and exploitation in swarm methods [45]. As for the MKP, these are single algorithms, and any of them could enter the framework studied here as a portfolio member. Table 1 summarizes the lines of work reviewed in this section against the present framework.

## 3. The Technique: DRL-Orchestrated Metaheuristic Portfolio

Table 2 lists the notation used in this section.

### 3.1. Problem Definition

Given *n* items with profits $p_{j}>0$, *m* resources with capacities $c_{i}$, and consumptions $r_{ij}\geq 0$, the MKP is

(1) $$\max\;f\left(x\right)=\sum_{j=1}^{n}p_{j}x_{j}\;\mathrm{s.t.}\;\sum_{j=1}^{n}r_{ij}x_{j}\leq c_{i}\;\;\left(i=1,\ldots ,m\right),\;x\in {\left\{0,1\right\}}^{n}.$$

Infeasible candidates are repaired by a greedy randomized constructive step followed by variable neighborhood descent and iterated local search; the operator is applied only to infeasible candidates, and feasible ones enter the population as generated, which matters on the loosest instances (Section 5.5). Quality is reported as the relative gap to the reference value, $\mathrm{GAP}=\left(f^{\mathrm{ref}}-f\left(x\right)\right)/f^{\mathrm{ref}}\times 100$, with $f^{\mathrm{ref}}$ the instance reference value. The second domain is the Set Covering Problem (SCP). Given *m* rows, *n* columns with costs $w_{j}>0$, and a binary matrix with $a_{ij}=1$ when column *j* covers row *i*, the SCP is

(2) $$\min\;f\left(x\right)=\sum_{j=1}^{n}w_{j}x_{j}\;\mathrm{s.t.}\;\sum_{j=1}^{n}a_{ij}x_{j}\geq 1\;\;\left(i=1,\ldots ,m\right),\;x\in {\left\{0,1\right\}}^{n}.$$

The environment maximizes, so the fitness it receives for the SCP is −f(x). Infeasible candidates are repaired by a greedy randomized completion of the cover followed by the removal of redundant columns, most expensive first, and quality is reported as $\mathrm{GAP}=\left(f\left(x\right)-f^{\mathrm{ref}}\right)/f^{\mathrm{ref}}\times 100$, with $f^{\mathrm{ref}}$ the optimal or best-known value of the instance. Both problems enter the environment through a problem factory that supplies the instance reader, constructive and repair operators, and fitness, so that the decision process defined next is the same for the two domains.

### 3.2. The Hyper-Heuristic as a Markov Decision Process

At every step *t*, the hyper-heuristic decides which portfolio metaheuristic runs for a quantum of *Q* population iterations. This control problem is modeled as a Markov decision process

(3) M=(S,A,P,R,γ),

with state space $S\subseteq {\left[0,1\right]}^{d}$, action space A={0,1,…,k−1} (one index per metaheuristic), transition kernel P, reward R, and discount γ∈(0,1]. Each episode disposes of a budget *B* of objective evaluations and ends when it is exhausted, after at most B/(Q·N) decisions for population size *N* (Section 4.2). The portfolio comprises k=7 population-based metaheuristics, a family widely applied to binary combinatorial problems such as the knapsack [46,47], chosen to cover five algorithmic families spanning three biological metaphors (Table 3); this diversity is deliberate, since a selector only adds value when the portfolio members respond differently to the search state. All methods maintain a fixed population of N=100 individuals, the condition that enables population transfer.

The above Markov decision process along with the portfolio of Table 3, the state of the next subsection, the population transfer transition of Equation (4), and the reward are a single shared framework common to every technique in this work. The three techniques studied here differ only in how the action space A is instantiated, in increasing order of richness, and all are trained with the same algorithm and compared under the same protocol:

- Selection: A discrete action $a_{t}\in \left\{0,\ldots ,k-1\right\}$ that chooses which metaheuristic runs the next quantum.
- Selection and configuration: A hybrid action that additionally tunes the chosen method with a configuration vector $\kappa \in {\left[0,1\right]}^{3}$.
- Heterogeneous co-evolution: A continuous simplex allocation that runs several metaheuristics in parallel on the shared pool.

The state, transition, and reward defined next are common to all three.

**Figure 1:**
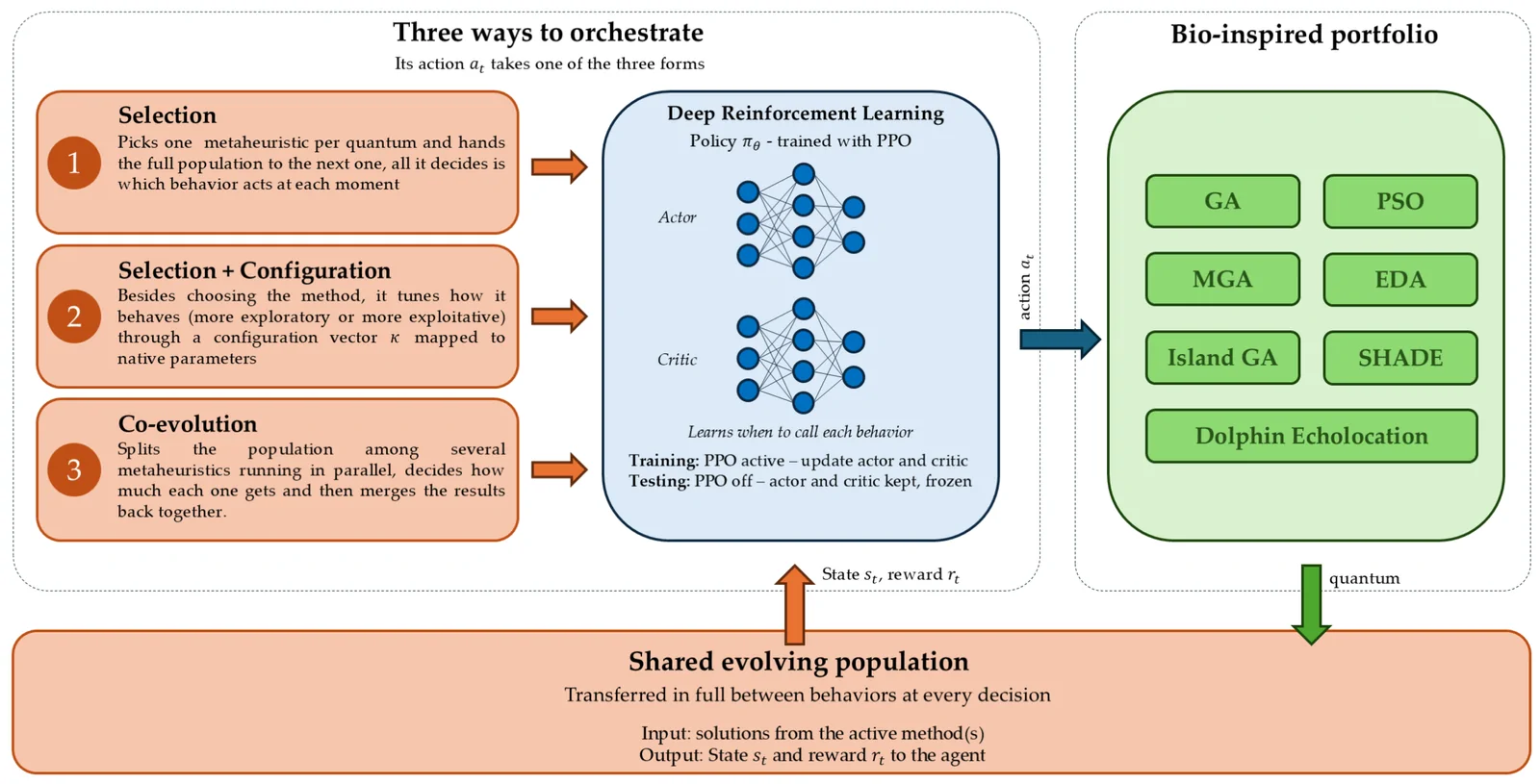
Conceptual overview of the framework. Each arrow takes the color of the block it leaves, and its label names the quantity that flows.

Figure 1 summarizes this shared framework: the agent’s action takes one of the three forms above and drives the bio-inspired portfolio over a single shared population.

The per-quantum dynamics are: (1) the agent observes $s_{t}$ and selects $a_{t}∼\pi_{\theta }\left(\cdot |s_{t}\right)$; (2) the selected metaheuristic, denoted ${\mathrm{MH}}_{a_{t}}$, evolves the population for *Q* iterations,

(4) $$P_{t+1}={\mathrm{MH}}_{a_{t}}\left(P_{t}\right);$$

(3) the incumbent is updated and evaluations are accumulated, $e_{t+1}=e_{t}+\Delta e_{t}$; and (4) the reward $r_{t}$ is collected, with the episode terminating when $e_{t+1}\geq B$. Equation (4) expresses the full population transfer mechanism: the entire population is handed to the incoming metaheuristic without re-initialization. The internal state of each method (the adaptive memory of SHADE, the velocities of PSO) is rebuilt at every quantum; the population is the only carrier of information across switches, which structurally favors methods for which the strength lies in population dynamics and penalizes those that rely on accumulated internal state, even when the same method is selected twice in a row. This is shared by every selection strategy compared in Section 5.

**Figure 2:**
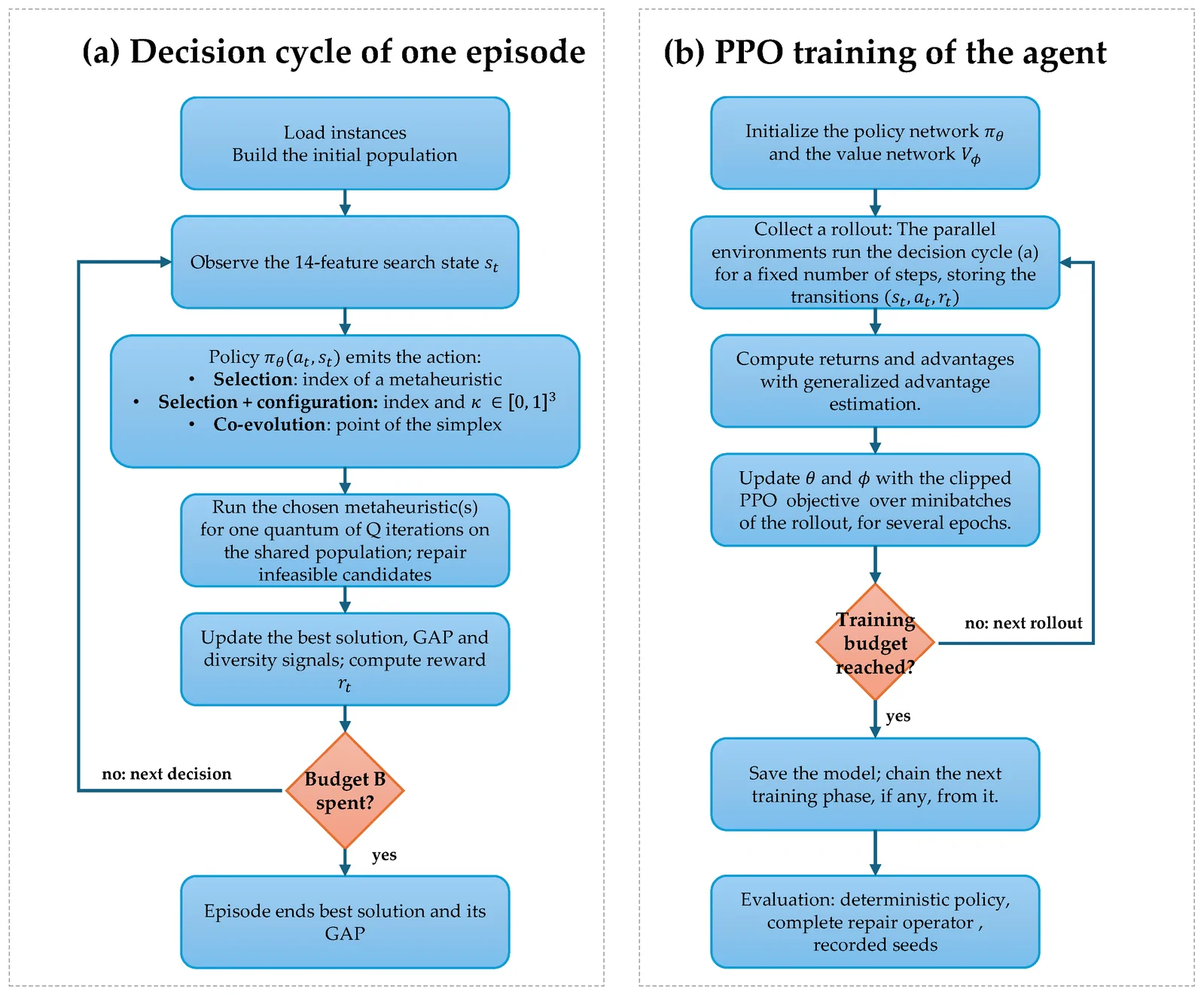
Flowchart of the decision cycle of one episode (**a**) and of PPO training (**b**).

Figure 2 summarizes the two loops of the method: the decision cycle of an episode, which the environment runs; and the PPO training loop that wraps it.

### 3.3. State Space

The state is a fixed-dimension vector with d=7+k (here, d=14), built by an extractor $\psi :\;\mathrm{metrics}\to {\left[0,1\right]}^{d}$:

(5) $$s_{t}=(\;\underset{7\;\mathrm{search}\;\mathrm{features}}{\underset{︸}{s_{t}^{\mathrm{ISM}},\;D_{t},\;H_{t},\;s_{t}^{\mathrm{col}},\;\iota_{t},\;\nu_{t},\;\tau_{t}}},\;\underset{\mathrm{one-hot},\;k}{\underset{︸}{e(a_{t-1})}}\;).$$

The components are: the stagnation signal $s_{t}^{\mathrm{ISM}}=\min\left({\mathrm{ISM}}_{t}/{\mathrm{ISM}}_{\max},\;1\right)$, where ${\mathrm{ISM}}_{t}$ (iterations since the last improvement) counts iterations without incumbent improvement (${\mathrm{ISM}}_{\max}=200$); the population diversity as mean per-gene binary entropy,

(6) $$D_{t}=\frac{1}{n}\sum_{j=1}^{n}H_{b}\left(q_{j}\right),\;H_{b}\left(q\right)=-q\log_{2}q-\left(1-q\right)\log_{2}\left(1-q\right),$$

where $q_{j}$ is the frequency of gene *j* in the population; the mean normalized Hamming distance to the incumbent, $H_{t}=\frac{1}{Nn}\sum_{i=1}^{N}d_{H}\left(x^{\left(i\right)},x^{*}\right)$; the diversity collapse signal $s_{t}^{\mathrm{col}}=\min\left({\mathrm{iters}}_{<90\%}/{\mathrm{col}}_{\max},\;1\right)$ with ${\mathrm{col}}_{\max}=100$; the recent improvement indicator $\iota_{t}$; the offspring acceptance rate $\nu_{t}$; the budget fraction $\tau_{t}=\min\left(e_{t}/B,\;1\right)$; and the one-hot encoding of the previous action. No component contains MKP-specific quantities (profits, consumption, capacities): the state is domain-agnostic by construction within binary representations, which enables the evaluation of a trained policy across instance sizes and, as Section 5.4 shows, across binary domains. The process is formally a partially observable MDP, since transitions depend on the full population while the agent observes this seven-feature summary; the cumulative signals ($s^{\mathrm{ISM}}$, $s^{\mathrm{col}}$) partially compensate, and the policy network is memoryless.

### 3.4. Selection: Discrete Action

In the base technique, the action is the discrete choice $a_{t}\in A=\left\{0,1,\ldots ,k-1\right\}$: at each decision, the agent selects which portfolio metaheuristic runs the next quantum and the chosen method is executed with its default configuration. This is the leanest instantiation of the shared framework and serves as the reference against which the richer action spaces below are measured.

Algorithm 1 states the resulting decision step.
**Algorithm 1** Selection decision step (discrete action)**Require:**  shared population $P_{t}$; action $a_{t}\in \left\{0,\ldots ,k-1\right\}$; quantum *Q*  1:  $\theta \leftarrow \theta_{a_{t}}^{\mathrm{def}}$▹ default configuration of the chosen method  2:  evolve $P_{t+1}\leftarrow {\mathrm{MH}}_{a_{t}}^{\;\theta }\left(P_{t}\right)$ for *Q* iterations▹ full population transfer  3:  repair the offspring in $P_{t+1}$  4:  **return** $P_{t+1}$

### 3.5. Joint Selection and Configuration: Hybrid Action

The extension that defines the technique replaces the discrete action with a hybrid pair

(7) $$\tilde{a}=\left(a,\kappa \right)\in \tilde{A},\;\tilde{A}=\left\{0,\ldots ,k-1\right\}\times {\left[0,1\right]}^{q},$$

where *a* selects the metaheuristic and $\kappa \in {\left[0,1\right]}^{q}$ is a vector of abstract knobs with q=3: $\kappa =(\kappa_{e},\;\kappa_{x},\;\kappa_{p})$, where $\kappa_{e}$ controls exploration intensity, $\kappa_{x}$ exploitation intensity, and $\kappa_{p}$ population pressure. These are the three quantities through which the parameter control literature characterizes a population-based metaheuristic [49,50]: every native hyperparameter that affects the exploration–exploitation balance of a method maps onto one of them, so the policy acts on a single three-dimensional space where interpretation is the same for every action, and the heterogeneity of the seven native parameter spaces is absorbed by the translation maps introduced next. Each metaheuristic *a* has its own heterogeneous hyperparameter space $\Theta_{a}$; a translation map defined outside the network,

(8) $$\varphi_{a}:\;{\left[0,1\right]}^{q}⟶\Theta_{a},$$

converts the knobs into native parameters, and the transition of Equation (4) becomes

(9) $$P_{t+1}={\mathrm{MH}}_{a_{t}}^{\;\varphi_{a_{t}}\left(\kappa_{t}\right)}\left(P_{t}\right).$$

Pure selection is the particular case in which $\varphi_{a}$ is constant, $\varphi_{a}\left(\kappa \right)\equiv \theta_{a}^{\mathrm{def}}$; the hybrid formulation strictly generalizes it. As a worked example, for PSO the map controls inertia and the cognitive and social coefficients:

(10) $$\begin{array}{cccc} \omega (\kappa ) & =0.40+0.50\;\kappa_{e} & \; & \in [0.40,\;0.90], \end{array}$$

(11) $$\begin{array}{cccc} c_{1}\left(\kappa \right) & =1.00+1.20\;\kappa_{x} & \; & \in [1.00,\;2.20], \end{array}$$

(12) $$\begin{array}{cccc} c_{2}\left(\kappa \right) & =1.00+1.20\;\kappa_{p} & \; & \in [1.00,\;2.20], \end{array}$$

so inertia grows with exploration, attraction to the personal best with exploitation, and attraction to the global best with population pressure. The remaining six maps follow the same linear pattern and are listed in Appendix A; default knob levels reproduce the literature settings of each method.

The policy emits the pair from one network body through two heads:

(13) $$\pi_{\theta }\left(\tilde{a}|s\right)=\pi_{\theta }^{\mathrm{sel}}\left(a|s\right)\;\cdot \;\pi_{\theta }^{\mathrm{cfg}}\left(\kappa |s\right),$$

where the selection head produces *k* logits and the configuration head produces the *q* knobs, discretized into eleven levels per coordinate for training stability, giving an action space of type MultiDiscrete([7,11,11,11]), that is, $7\times 11^{3}$ = 9317 joint actions. Each additional knob would multiply that number by eleven, beyond what the training budget of this study can cover, which is why *q* is kept at three; whether a larger or a learned configuration space would improve the joint agent is left open (Section 7). The hyperparameters of the metaheuristic are outputs of the network. One structural property of this factorization must be noted: within a single decision, κ is conditioned on the state but not on the sampled method *a*; per-method configuration behavior arises through the state, which encodes the previous action, rather than through within-step conditioning. An autoregressive policy would remove this limitation and is left as future work.

Algorithm 2 states the resulting decision step.
**Algorithm 2** Joint selection-and-configuration decision step (hybrid action)**Require:**  shared population $P_{t}$; action ${\tilde{a}}_{t}=\left(a_{t},\kappa_{t}\right)$ with $\kappa_{t}\in {\left[0,1\right]}^{q}$; quantum *Q*  1:  $\theta \leftarrow \varphi_{a_{t}}\left(\kappa_{t}\right)$▹ translation map: abstract knobs → native parameters  2:  evolve $P_{t+1}\leftarrow {\mathrm{MH}}_{a_{t}}^{\;\theta }\left(P_{t}\right)$ for *Q* iterations▹ full population transfer  3:  repair the offspring in $P_{t+1}$  4:  **return** $P_{t+1}$

### 3.6. Heterogeneous Co-Evolution: Simplex Allocation

The third formulation generalizes the discrete selection to a simultaneous allocation across the portfolio, so that several metaheuristics evolve in parallel on a shared pool rather than one per step. At each decision the policy emits a continuous vector $z_{t}\in {\left[-10,10\right]}^{k}$ that is mapped to a point on the probability simplex through a softmax,

(14) $$\alpha_{t}=\mathrm{softmax}\left(z_{t}\right)\in \Delta^{k-1},\;\sum_{a=0}^{k-1}\alpha_{t,a}=1,$$

where $\alpha_{t,a}$ is the share of the pool assigned to metaheuristic *a*. An activation floor η=0.10 defines the active set $K_{t}=\left\{\;a:\alpha_{t,a}\geq \eta \;\right\}$, and the allocation is renormalized over $K_{t}$ to obtain ${\bar{\alpha }}_{t}$. The shared pool of *N* individuals is then partitioned into demes of size $N_{t,a}=\left\lfloor {\bar{\alpha }}_{t,a}\;N\right\rfloor$ for each $a\in K_{t}$. Every active metaheuristic evolves its own deme for one quantum,

(15) $$P_{t+1}^{\left(a\right)}={\mathrm{MH}}_{a}\;\left(P_{t}^{\left(a\right)}\right),\;a\in K_{t},$$

and a fusion operator μ re-unites the demes into the shared pool and re-partitions it at the next decision, so the population is transferred as a whole across steps exactly as in the base selection dynamics.

To expose the per-method search state, the observation is extended to $d_{C}=7+3k=28$ dimensions: the seven global features of the base state plus a triplet per metaheuristic (its relative improvement, deme diversity, and mean allocation). The action is realized as a $\mathrm{Box}({\left[-10,10\right]}^{k})$ space with an internal softmax. The base selection Markov decision process is recovered as the special case in which $\alpha_{t}$ collapses to a one-hot vertex of the simplex, that is, a single active metaheuristic per step; the co-evolution formulation thus contains both the pure selection and the concurrent-allocation regimes as limits of the same simplex policy.

Algorithm 3 states the resulting decision step.
**Algorithm 3** Heterogeneous co-evolution decision step (simplex allocation)**Require:**  shared population $P_{t}$ of size *N*; action $z_{t}\in {\left[-10,10\right]}^{k}$; floor η; quantum *Q*  1:  $\alpha \leftarrow \mathrm{softmax}(z_{t})$▹ point on the simplex $\Delta^{k-1}$  2:  $K\leftarrow \{\;a:\alpha_{a}\geq \eta \;\}$; renormalize α over *K* to get $\bar{\alpha }$  3:  partition $P_{t}$ into demes ${\left\{P^{\left(a\right)}\right\}}_{a\in K}$ with $|P^{\left(a\right)}|=\left\lfloor {\bar{\alpha }}_{a}\;N\right\rfloor$  4:  **for** each a∈K **in parallel do**  5:     $P^{\left(a\right)}\leftarrow {\mathrm{MH}}_{a}\;\left(P^{\left(a\right)}\right)$ for *Q* iterations  6:  **end for**  7:  $P_{t+1}\leftarrow \mu \;\left({⋃}_{a\in K}P^{\left(a\right)}\right)$▹ fuse demes into the shared pool  8:  repair $P_{t+1}$  9:  **return** $P_{t+1}$

### 3.7. Reward

The reward combines three signals with weights $w_{I},w_{S},w_{D}\geq 0$:

(16) $$r_{t}=w_{I}\;r_{t}^{I}+w_{S}\;r_{t}^{S}+w_{D}\;r_{t}^{D}.$$

The improvement term is the scale-invariant relative gain of the incumbent,

(17) $$r_{t}^{I}=\frac{f_{t}^{*}-f_{t-1}^{*}}{\left|f_{t-1}^{*}\right|}\;\mathrm{if}\;\left|f_{t-1}^{*}\right|>\varepsilon ,\;r_{t}^{I}=0\;\mathrm{otherwise};$$

the stagnation penalty grows quadratically, $r_{t}^{S}=-\lambda_{S}{\left({\mathrm{ISM}}_{t}/{\mathrm{ISM}}_{\max}\right)}^{2}$; and the diversity-recovery bonus is

(18) $$r_{t}^{D}=\left\{\begin{array}{cc} 0.5, & \mathrm{if}\;\mathrm{diversity}\;\mathrm{was}\;\mathrm{collapsed}\;\mathrm{and}\;D_{t}>D_{t-1},\\ 0, & \mathrm{otherwise}. \end{array}\right.$$

Reference weights: $w_{I}=1.0$, $w_{S}=0.3$, $w_{D}=0.2$, $\lambda_{S}=0.5$.

### 3.8. Bi-Level Objective and Learning Algorithm

The technique chains two optimization levels. At the lower level, each metaheuristic maximizes *f* over ${\left\{0,1\right\}}^{n}$; at the upper level, the agent seeks the policy parameters that maximize the expected quality of the best solution produced when the budget is exhausted:

(19) $$\theta^{*}=\arg\max_{\theta }\;E_{\tau ∼\pi_{\theta }}\;\left[\;f\left(x_{\mathrm{final}}^{*}\right)\right].$$

The upper level is solved with PPO [10] in Stable-Baselines3 version 2.8 [51]: separate policy and value networks of sizes 256/128 and 256/256, learning rate $3\times 10^{-4}$, clip range 0.2, generalized advantage estimation with λ=0.95, discount γ=0.99, entropy coefficient 0.05, 10 optimization epochs per update with minibatches of 64, and rollouts of 32 steps per environment from 14 parallel environments (448 transitions per update). The selection agent uses a single categorical head over the seven methods; over the MultiDiscrete space of the joint agent the policy factorizes into four categorical distributions, and the co-evolution agent uses a diagonal Gaussian over its box space. The per-step reward of Equation (16) acts as a surrogate for the bilevel objective of Equation (19); Section 4 and Section 6 discuss when this surrogate remains informative: it saturates at generous budgets, and its mass concentrates on the first decision of the episode.

Algorithm 4 summarizes the training loop, which is identical for the three techniques and differs only in the sampled action and the technique-specific population update (Algorithms 1–3).
**Algorithm 4** PPO training of the orchestration policy (shared by the three techniques)**Require:** portfolio ${\left\{{\mathrm{MH}}_{a}\right\}}_{a=0}^{k-1}$; training instances $I_{\mathrm{train}}$; budget *B*, quantum *Q*, population *N*; discount γ, generalized advantage estimation (GAE) parameter λ, clip ϵ, epochs *E*, minibatch *M*  1:  initialize policy $\pi_{\theta }$ and value $V_{\phi }$ (separate multilayer perceptrons, MLPs)  2:  **repeat**  3:     D←∅  4:     **for** each parallel environment **do**  5:           sample $I∼I_{\mathrm{train}}$; initialize and repair the population; observe $s_{0}$  6:           **for** t=0,1,… until *B* evaluations are consumed **do**  7:                 sample action $u_{t}∼\pi_{\theta }\left(\cdot |s_{t}\right)$▹$a_{t}$, ${\tilde{a}}_{t}$, or $z_{t}$, per technique  8:                 apply the technique’s decision step▹ Algorithms 1–3  9:                 observe $s_{t+1}$ and reward $r_{t}$; store $\left(s_{t},u_{t},r_{t}\right)$ in D10:           **end for**11:     **end for**12:     compute returns ${\hat{R}}_{t}$ and GAE advantages ${\hat{A}}_{t}$ over D13:     **for** epoch =1,…,E **do**14:           **for** each minibatch of size *M* from D **do**15:                 update θ by maximizing the clipped surrogate $L^{\mathrm{CLIP}}\left(\theta \right)$ with an entropy bonus16:                 update ϕ by minimizing ${\left(V_{\phi }\left(s_{t}\right)-{\hat{R}}_{t}\right)}^{2}$17:           **end for**18:     **end for**19:  **until** the training-step budget is exhausted20:  **return** policy $\pi_{\theta }$

## 4. Experimental Methodology

The experimental regime of this study was not chosen a priori; it is the product of a documented diagnostic study on a smaller benchmark. That study, on the cb7 set (n=100 items) under a generous budget, showed that the benchmark was solved by the budget itself: the whole portfolio reached near-optimal solutions and no selector, learned or blind, had room to separate. This finding shaped the present design: a larger instance set (cb9, n=500 items) that does not saturate at a moderate budget and a tighter evaluation budget that keeps the comparison informative. The three techniques are evaluated under this regime. Section 5.6 repeats the cb7 study under the protocol of this paper and reports both benchmarks side by side.

### 4.1. Instances and Reference Values

Experiments use the Chu and Beasley set cb9 from OR-Library [33]: thirty instances with n=500 items and m=30 knapsack constraints, the largest of the family and the regime in which the selection problem is non-trivial. The multidimensional knapsack problem was chosen as the primary benchmark because it is a canonical NP-hard binary problem with a standard instance library, published best-known values that make the GAP measurable and a hyper-heuristic literature on the same instances [36]; the Set Covering Problem of Section 5.4 was added under the same protocol to test whether the conclusions depend on the domain. The instances were transcribed from the official OR-Library files and checked against them. Reference values are the best-known values reported by Lai et al. in 2018 [11], obtained with a two-phase tabu-evolutionary algorithm, incorporating the three later improvements (at most +0.014%, on instances 30.500-03, -04, and -18) reported by Xu et al. [12] (Appendix B); to our knowledge, no other best-known value of the set has changed since. The GAP is measured against these references. The thirty instances are organized by the constraint tightness ratio α∈{0.25,0.50,0.75} (ten instances per value). This parameter governs how restrictive the resource capacities are, and hence the difficulty of the instance.

### 4.2. Protocol

The thirty cb9 instances are evaluated under five-fold cross-validation. The folds are stratified by constraint tightness: each fold holds six instances, two from each tightness group (α=0.25, 0.50, 0.75), so every fold covers the three groups. For each fold, the agent is trained from scratch on the twenty-four remaining instances and evaluated on the six it never saw; every instance is evaluated exactly once, by a model that did not see it during training. Training runs four chained phases of 5376 timesteps each (21,504 timesteps per fold), with the learner and the environment seeded per phase so that the campaign is reproducible. Runs use a fixed budget of B=25,000 evaluations, quantum Q=25, and population N=100, that is, at most ten decisions per episode: a quantum consumes at least Q·N evaluations. For MGA, the local search evaluates additional candidates, spending about 2900, so an episode that selects it repeatedly ends after nine decisions with the same budget consumed. These values were fixed before training: *B* is the budget at which cb9 separates the stronger from the weaker members without converging, and *Q* gives each decision a measurable effect within it; Section 5.5 examines how the comparison changes with both. Every evaluation reported in the results uses the complete repair operator.

The repair operator is shared by every strategy compared below. It belongs to the problem, and the environment applies it identically whichever strategy produces the candidate solutions, so differences between strategies are attributable to how they generate and orchestrate populations and not to the repair. To measure the contribution of the operator on its own, the evaluation also includes a repair-only baseline that uses no population and no metaheuristic: it draws a random solution, applies the repair operator, keeps the best result, and repeats until the same evaluation budget is exhausted in the manner of a greedy randomized adaptive search procedure (GRASP) multi-start [52]. The baseline receives no less repair effort than any portfolio member (1.00 repairs per counted evaluation; the portfolio members range from 0.62 for Island GA, MGA and GA to 0.999 for PSO and SHADE) and, being seeded as the environment is, starts each paired scenario from the same initial solution as the other strategies.

Each held-out instance is evaluated with twenty seeds, drawn once at random before any evaluation and recorded with the results, so that all strategies run exactly the same 30×20=600 instance–seed scenarios. The comparison covers fifteen strategies inside the same quantum-based environment: the three trained agents (selection, joint selection-and-configuration, and co-evolution, each a deterministic policy), each of the seven metaheuristics run alone, uniform random selection, round-robin, and three control strategies, namely: the repair-only baseline described above; an untrained control, a network with the architecture of the selection agent that receives no PPO update and is queried in the same deterministic mode, with a different random initialization in every run so that its row describes the distribution of untrained performance; and the selection agent retrained on the portfolio without PSO and Dolphin (Section 5.3). Quality is aggregated as the GAP against the best-known values (Appendix B), averaged over the 600 runs of each strategy; dispersion is the standard deviation over the same runs, and the dispersion of the five fold means is reported separately. Pairwise differences between the selection agent and each other strategy are assessed with a two-sided paired Wilcoxon signed-rank test over the 600 scenarios, and the effect size is reported as the Vargha–Delaney ${\hat{A}}_{12}$ statistic [53], with the magnitude thresholds recommended for randomized algorithms by Arcuri and Briand [54]. The Wilcoxon test is paired by scenario, whereas ${\hat{A}}_{12}$ compares the two pooled distributions of runs of the strategies involved. As a robustness check, the cb9 evaluation was repeated with a second, independent set of twenty seeds: the ranking of the thirteen strategies involved is the same except that the two blind selectors, for which the means differ by less than 0.002 points, exchange places, and no competitive strategy differs by more than 0.006 GAP points from the values reported below. The instances, the fold compositions, the registry of seeds, the per-run results, the trained models and the source code of the framework are available in the public repository cited in the Data Availability Statement. The same protocol, with the domain entering only through the problem factory, is applied to the Set Covering Problem in Section 5.4.

## 5. Results

### 5.1. Training Dynamics

Five selection agents were trained on cb9, one per fold, each for 21,504 timesteps in four chained phases with 14 parallel environments (about 2130 episodes per fold). The optimization-side diagnostics are consistent across folds. The explained variance of the value function ends between 0.655 and 0.673, so the critic fits the returns to a similar degree in every fold. The entropy of the stochastic training policy ends between 0.94 and 0.97 of its maximum lnk with k=7: the entropy bonus keeps the policy that generates the training data close to uniform, and every method receives between 11% and 18% of the training decisions, PSO the fewest in every fold.

**Figure 3:**
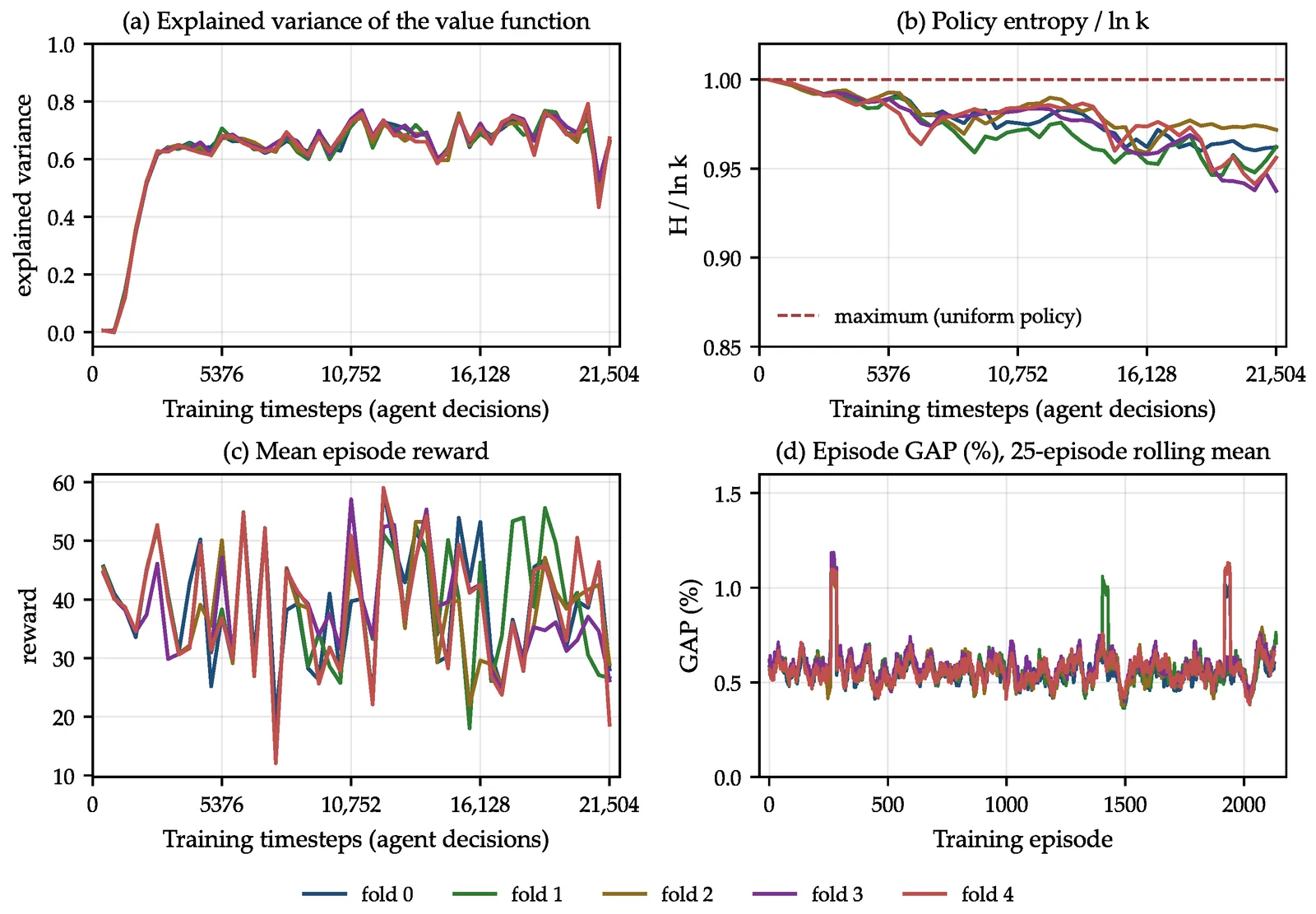
Training diagnostics of the five cb9 selection agents, one curve per fold. In (**a**–**c**), the horizontal axis counts training timesteps, one per agent decision (a quantum of Q=25 iterations on one of the fourteen parallel environments) up to the 21,504 of the reported model; (**d**) is indexed by training episode.

**Figure 4:**
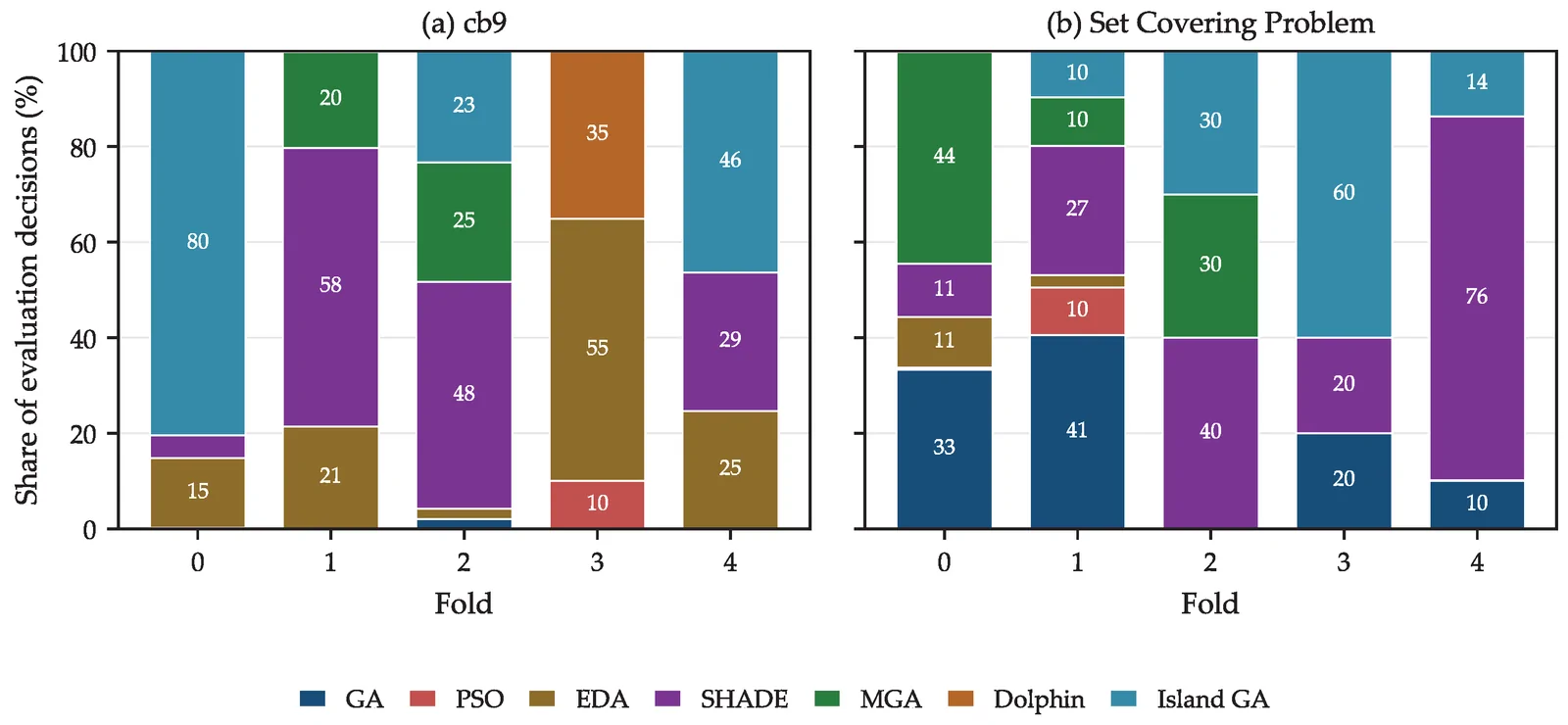
Composition of the deterministic policies at evaluation, per fold: (**a**) cb9 and (**b**) the Set Covering Problem. Bars give the share of evaluation decisions assigned to each metaheuristic; labels below 8% are omitted.

The deterministic policy used at evaluation, which takes the most probable action in each state, is far from uniform and differs from fold to fold. In fold 0, it assigns 80% of its decisions to Island GA; in folds 1 and 2, SHADE leads (58% and 48%), combined with MGA and EDA in one case and with MGA and Island GA in the other; in fold 4, the decisions are shared between Island GA (46%), SHADE (29%), and EDA (25%); in fold 3 the policy relies on EDA (55%), Dolphin (35%), and PSO (10%). Over the 5999 evaluation decisions of the five folds (one run ended after nine decisions), Island GA receives 30.1%, SHADE 27.9%, EDA 23.5%, MGA 9.0%, Dolphin 7.1%, PSO 2.0%, and GA 0.5%. Two features of these policies matter for the comparison below: the two methods that collapse as standalone solvers are selected almost exclusively in a single fold (all 120 PSO decisions and 422 of the 423 Dolphin decisions fall in fold 3), and that fold is the one in which the agent performs worst. Figure 3 shows the four diagnostics over training for the five folds, while Figure 4 shows the composition of the resulting deterministic policies on cb9 and the second domain of Section 5.4.

### 5.2. Comparative Evaluation on cb9 (RQ1)

Table 4 ranks the fifteen strategies over the 600 runs of the cross-validation protocol, and Table 5 disaggregates the same runs by fold. The field is compact: eleven of the fifteen strategies lie between 0.34% and 0.47% of mean GAP, and only PSO and Dolphin (7.9% and 6.4%) and the two controls that use either no learned policy or no metaheuristic at all (untrained agent 1.04% and repair-only 1.01%, respectively) fall outside that band.

Two columns of Table 4 require further analysis: the GAP column orders the strategies by mean solution quality, while the SD column measures the dispersion over the 600 runs of each strategy. Because every strategy runs the same instance–seed scenarios, more than 90% of that dispersion for each of the eleven competitive strategies consists of the spread of difficulty across the thirty instances rather than run-to-run noise. Their SDs are similar (0.21 to 0.27, and 0.37 for the selection-and-configuration agent) and larger than the difference between any two of their means. These differences are resolved because the comparison is paired by scenario; this is why the table also reports the paired Wilcoxon *p*-value and the effect size for every row. A standard deviation large relative to the mean identifies a strategy that fails on some runs rather than one that is uniformly worse: the untrained control (SD 3.35 for a mean of 1.04%), Dolphin (8.07) and PSO (9.77) are of that kind, whereas no run of the selection or co-evolution agent exceeds a GAP of 1.13%. Island GA alone leads with 0.347%, followed by the co-evolution agent (0.362%), MGA alone (0.371%), the selection agent retrained on the five-method portfolio (0.388%), and the selection agent (0.390%). The selection agent is ahead of every strategy that does not learn from the search state: it improves on round-robin (0.417%) and uniform random selection (0.418%) in four of the five folds and the paired test rejects equality with $p<10^{-12}$ in both cases, although the effect size is negligible (${\hat{A}}_{12}=0.55$) because the margin is 0.03 points on runs for which the standard deviation is 0.24. It is behind the strongest single method; Island GA beats it by 0.043 points ($p<10^{-25}$), a difference that the paired test detects on 600 scenarios but for which the effect size remains negligible (${\hat{A}}_{12}=0.445$); MGA is also ahead ($p<10^{-4}$). The co-evolution agent, which allocates the population among several methods at each decision instead of committing it to one, ends 0.015 points behind Island GA and 0.027 points ahead of the selection agent ($p<10^{-8}$). No learned policy surpasses the best member of the portfolio on this benchmark; the two competitive learned policies come within 0.015 and 0.043 points of it without being told which member it is, and do so ahead of the blind selectors.

The per-fold view explains the margin to Island GA. The selection agent beats Island GA in fold 0 (0.398% against 0.402%) and trails it by 0.01 to 0.06 points in folds 1, 2 and 4; fold 3, the only fold for which the policy selects Dolphin and PSO (Section 5.1), is its worst (0.454% against 0.328%) and accounts for most of the mean difference. The dispersion of the fold means is similar for the eleven competitive strategies ($\sigma_{\mathrm{folds}}$ between 0.03 and 0.05), with one exception: the joint selection-and-configuration agent, for which the action space is larger by a factor of $11^{3}$ (Section 3), ranks tenth (0.460%) because its fold 0 policy collapses onto a weak configuration (0.712%) while its other four folds lie between 0.37% and 0.42%; at this training budget it does not improve on the blind selectors.

The three controls place the numbers in context. The repair-only baseline reaches 1.007% and loses to the selection agent in 595 of the 600 scenarios (${\hat{A}}_{12}=0.77$): the operator on its own brings the GAP to about 1%, and the portfolio and its orchestration account for the reduction below 0.4%. The untrained agent, evaluated with a fresh random initialization in every run, reaches 1.042% with a standard deviation of 3.35, fourteen times that of the trained agent; 21 of its 600 runs end with a GAP above 5% (the worst above 20%), against none of the runs of the trained agent (maximum 1.12%), and it selects PSO in 11.4% and Dolphin in 12.2% of its decisions, against 2.0% and 7.1% for the trained policies. The trained agent wins 345 of the 600 paired scenarios ($p<10^{-5}$) with a negligible effect size (${\hat{A}}_{12}=0.548$), so learning does not shift the typical run but removes the catastrophic ones (Section 6). The agent retrained without PSO and Dolphin ties with the seven-method agent (0.388% against 0.390%); Section 5.3 examines this ablation together with the decision trace of the two methods.

**Figure 5:**
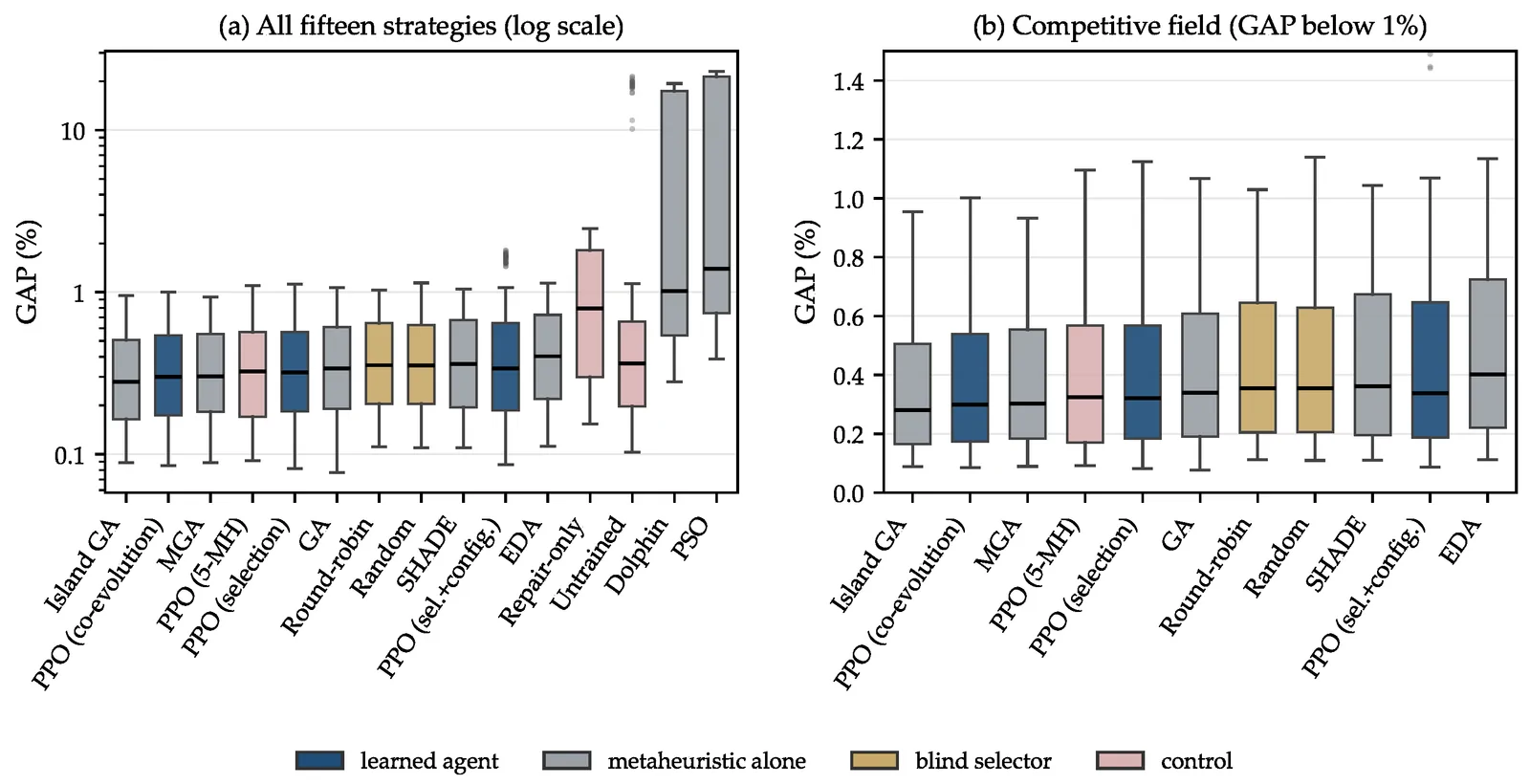
Distribution of the GAP over the 600 cross-validation runs of each strategy on cb9. (**a**) All fifteen strategies, logarithmic scale; (**b**) the eleven strategies with mean GAP below 1%, linear scale. Boxes: interquartile range and median; whiskers: 1.5× the interquartile range; dots: outliers; color: family of the strategy.

Figure 5 shows the distributions behind the means of Table 4. On the logarithmic panel, PSO and Dolphin lie more than an order of magnitude above the field (medians of 1.40% and 1.02%, third quartiles above 17%, one run in three above 5%); the repair-only baseline occupies the intermediate band (median 0.80%, one run in three above 1%); and the untrained control has a median close to that of the trained agent (0.36% against 0.32%) but a tail that reaches 21%, which is the signature of its catastrophic runs. On the linear panel, the eleven competitive strategies overlap: their medians lie between 0.28% (Island GA) and 0.40% (EDA), their third quartiles between 0.51% and 0.72%, and their whiskers end between 0.93% and 1.14%. Fewer than 1% of the runs of any of them exceed a GAP of 1%, with two exceptions: EDA (2.8% of its runs) and the joint selection-and-configuration agent (6.3%, all from its collapsed fold 0 policy, with a maximum of 1.81%). The difference between the selection agent and Island GA is a shift of a few hundredths of a point in the whole distribution, not a difference in the frequency of poor runs.

**Figure 6:**
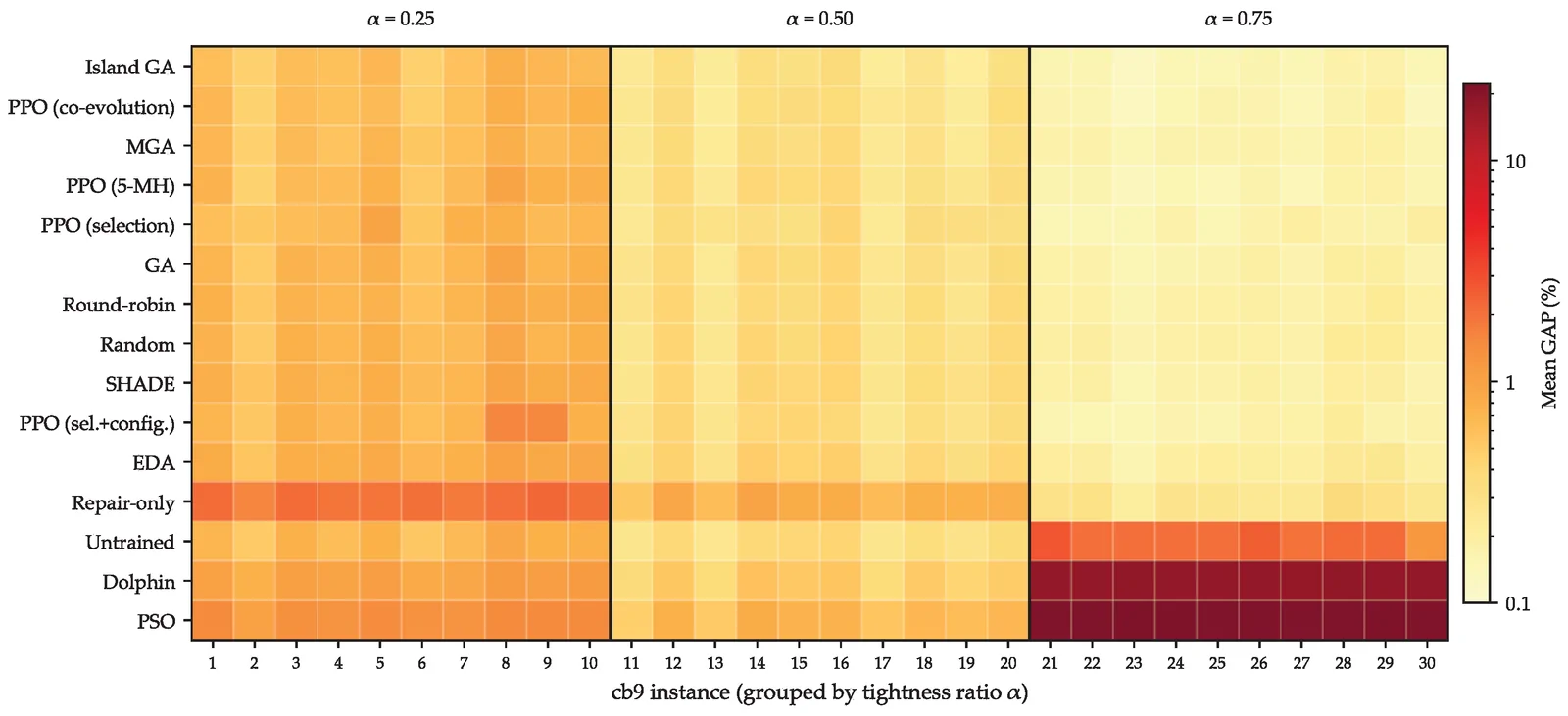
Mean GAP (%) per cb9 instance over the twenty seeds. Rows are strategies ordered by overall mean GAP, columns the thirty instances grouped by tightness ratio α; logarithmic color scale.

Figure 6 disaggregates the same runs by instance, and three patterns are visible. First, the tightness ratio orders the difficulty for every strategy: on the ten instances with α=0.25 the competitive strategies reach mean GAPs between 0.61% and 0.87%, on α=0.50 between 0.28% and 0.39%, and on α=0.75 between 0.15% and 0.20%; the repair-only baseline follows the same order (from 1.99% to 0.27%). Second, the collapse of PSO and Dolphin is confined to the α=0.75 block, where their mean GAP exceeds 17% on every instance, whereas on the other twenty instances they stay within a factor of 1.5 to 3 of Island GA; the untrained control degrades on the same block (2.09% mean, 10.5% of its runs above 5%, none elsewhere), because that is where a policy that selects PSO or Dolphin is punished. Third, within each block the rows of the competitive strategies are nearly indistinguishable by color: the selection agent attains a lower mean GAP than Island GA on 7 of the 30 instances (3, 9, 14, 15, 21, 22 and 29) and lower than round-robin on 22; the co-evolution agent beats Island GA on seven instances as well. No instance separates the competitive field by more than a few tenths of a point.

### 5.3. PSO and Dolphin: Decision Trace and Portfolio Ablation

PSO and Dolphin are the two portfolio members that collapse as standalone solvers (Table 4), and the deterministic policies of Section 5.1 rarely select them. Two analyses establish whether they contribute to the search when they are selected and what the portfolio loses without them.

The first analysis uses the training records of the five cb9 agents, which store, for every decision, the selected method and the value of the best solution of the episode before and after the quantum. The first decision of each episode is excluded, since any method improves the random initial population at that point; 95,372 decisions remain, distributed almost uniformly across the seven methods by the entropy bonus of PPO. Table 6 summarizes the outcome per method. PSO improves the best solution after 5.5% of its decisions and Dolphin after 10.6%, against 39.7% to 64.4% for the other five methods; after the fifth decision of an episode, PSO improves it in 3 of 6265 decisions and Dolphin in 6 of 7113. The two methods do not compensate with rare large gains: the 95th percentile of their improvement per decision is 0.08% and 1.15%, against about 15% for EDA and SHADE. The rates vary little across the four training phases and persist when each method is compared only with the others within the same episode (48.5 and 43.1 percentage points below them), so they are not explained by the states in which each method happened to be selected. The trained policies act on this evidence: at evaluation, they select PSO in 2.0% of their decisions and Dolphin in 7.1%; in four of the five folds PSO is never selected.

The second analysis retrains the selection agent on the portfolio without PSO and Dolphin under the protocol of Section 4.2: five folds, 21,504 timesteps per fold and the same twenty recorded seeds. The reduced portfolio reaches a mean GAP of 0.388% against 0.390% for the seven-method agent (p=0.31; Table 4). It is better in fold 3, where the seven-method policy had selected Dolphin (0.363% against 0.454%), and worse in folds 0 and 4. Within the reduced portfolio, selection without learning performs as well as the trained agent: uniform random selection reaches 0.380% and the untrained network 0.369%. The contribution of learning on cb9 is concentrated in avoiding the members that produce the worst results; once these are removed, the remaining selection problem is small. PSO and Dolphin are retained in the portfolio because the evidence that the agent learns to exclude them would disappear without them.

### 5.4. Second Domain: Set Covering Problem (RQ2)

RQ2 asks whether the same environment transfers to a different binary domain without changes to the state, the reward or the portfolio. The Set Covering Problem of Section 3.1 is the natural test: the solution is a binary vector, as in the MKP, but the objective is minimized, the feasibility condition is a covering rather than a capacity constraint, and feasible solutions are sparse (between 0.3% and 7% of the columns active depending on the set, against about 50% in the MKP). Only the problem factory changes: the instance reader; the randomized greedy constructive heuristic that builds the initial populations, with a restricted candidate list of the three columns of lowest cost per newly covered row; the repair operator; and the fitness function. Because the fitness is the negated cost, the relative improvement term of the reward becomes the relative cost reduction with no further change. The fourteen state features, the reward weights, the seven metaheuristics with their translation maps, the PPO hyperparameters and the regime B=25,000, Q=25, N=100 are those of cb9. The five training runs on the SCP end with an explained variance between 0.76 and 0.81 and an entropy between 0.86 and 0.89 of its maximum; Dolphin, not PSO, is the least sampled method during training (6% to 7% of the decisions).

The benchmark is the 65 OR-Library instances of Beasley’s sets 4, 5, 6, A–D and NRE–NRH [40,41], from 200 × 1000 to 1000 × 10,000 rows by columns and with matrix densities of 2%, 5%, 10%, and 20%. Density plays in the SCP the role that tightness plays in the MKP, so the five folds of thirteen instances are stratified by density. The reference values are the proven optima for sets 4–6, A–D, NRE and NRF and the best-known values of the literature for NRG and NRH. The same twenty recorded seeds give 65 × 20 = 1300 runs per strategy, over eleven strategies: the selection agent trained per fold with the cb9 procedure, the seven metaheuristics alone, the two blind selectors and the untrained control (Table 7 and Table 8).

On the SCP, the selection agent ranks first with a mean GAP of 1.529%, ahead of GA (1.599%, $p<10^{-3}$), MGA (1.626%, $p<10^{-5}$), SHADE (1.679%, p=0.012), and Island GA (1.702%, $p<10^{-10}$). Island GA, which leads cb9, places fifth here. The paired test rejects equality with every other strategy, with negligible effect sizes against the four strongest single methods (${\hat{A}}_{12}$ between 0.48 and 0.54) and small ones against the blind selectors (0.58 and 0.61). The SHADE comparison is the only one in which ${\hat{A}}_{12}$ falls below 0.5: SHADE attains the lower GAP in more scenarios (289 against 244, with 767 ties), and the agent’s advantage in the mean and in the signed-rank test comes from the larger size of SHADE’s losses. The fold view is more telling than the ranking: no single metaheuristic leads more than one fold (the agent leads folds 0 and 2, SHADE fold 1, MGA fold 3 and GA fold 4), and the agent is the only strategy that leads more than one fold; it never places below fourth, and has the best mean rank across folds (2.0, against 2.6 for GA and MGA, which also stay in the top four). Its deterministic policies again differ from fold to fold and concentrate on two or three of the four strongest members rather than on the leading method of the fold: MGA and GA in fold 0, GA and SHADE with a 10% share of PSO in fold 1, a mixture of SHADE, MGA and Island GA in fold 2, Island GA in fold 3 (where MGA leads), and SHADE in fold 4 (where GA leads). The fold 0 policy also shows the effect of MGA on the episode length: its episodes close after nine decisions with the same budget consumed (Section 4.2). Folds 2 and 3, which hold eight of the ten NRG and NRH instances, the largest of the benchmark (1000 × 10,000), are harder for every strategy (agent GAP 2.3% to 2.4% against 0.8% to 1.1% elsewhere). The untrained control reaches 1.903% and loses to the trained agent in 411 of the 1300 scenarios, ties in 788, and wins in 101 ($p<10^{-35}$). The transfer requires no change to the decision problem, and on a domain without a dominant portfolio member, the learned selection is the best strategy in the comparison.

### 5.5. Sensitivity to the Evaluation Budget and the Quantum

The protocol fixes B=25,000 and Q=25. Two additional measurements examine how the comparison depends on them. The first repeats the complete cb9 evaluation with half the budget, B=12,500, on the same 600 instance–seed scenarios, with the same twenty recorded seeds and the same trained models; nothing is retrained, so the agents act in episodes of five decisions instead of the ten they were trained on. Table 9 pairs the two budgets per strategy.

At half the budget the co-evolution and selection agents rank first and second (0.396% and 0.427%), round-robin third (0.447%), and the three methods that lead at B=25,000 fall to ninth, tenth and eleventh: Island GA 3.48%, GA 3.64% and MGA 5.29%. Every strategy is worse at the smaller budget, but by very different amounts: 0.02 to 0.04 points for the round-robin and EDA agents, against 3.1 to 4.9 points for the three GA-based methods. The loss is confined to the ten instances with α=0.75: there, Island GA, GA, and MGA end with a median GAP of 10.5%, 10.7% and 14.9% (between 85% and 100% of their runs above 5%), while EDA, SHADE, and the two agents stay between 0.17% and 0.22%. On the twenty instances with α≤0.50, Island GA remains the best strategy at B=12,500 (0.478% against 0.548% for the selection agent).

The cause is the interaction between the repair operator and the tightness of the instances. The operator is applied only to infeasible candidates (Section 3.1). On the loosest instances, a random candidate with half of the items selected is feasible in every case, with a GAP close to 38%, whereas on the tightest ones it is never feasible and is repaired at once to a GAP of about 4%. GA, MGA, and Island GA produce feasible offspring on loose instances and climb from that starting point by evolution alone, which takes more than 12,500 evaluations; SHADE and PSO binarize continuous vectors that are almost always infeasible (0.999 repairs per evaluation, Section 4.2) and receive the repair at every evaluation. A strategy that alternates methods inherits the fast start of the latter whichever member it selects afterwards. The advantage of the learned agents at the smaller budget is real within this environment, but rests on the composition of the portfolio as much as on the selection policy; round-robin obtains most of it.

The second measurement varies the quantum. On folds 0 and 2 (twelve instances), with three seeds of their own and six strategies (the selection agent, Island GA, MGA, GA, round-robin, and uniform random selection), the nine combinations of B∈{12,500;25,000;50,000} and Q∈{10,25,50} were evaluated with the same trained models. The budget has the expected effect on the agent (0.410%, 0.357% and 0.338% at Q=25, paired p≤0.007 between consecutive budgets), whereas the quantum has none that the data can resolve: at each budget the agent’s GAP differs by at most 0.023 points between quantum values, and no difference reaches significance (paired *p* between 0.10 and 0.92). Q=25 gives the lowest GAP of the agent at each of the three budgets. The agent ranks first in five of the nine cells and second in the other four: it leads the three cells at B=12,500, where the three single methods of the grid remain above 3.8% GAP, shares the lead with Island GA at B=25,000, and leads one cell at B=50,000, where Island GA leads the other two. For the blind selectors at B=12,500 a finer quantum helps (uniform random selection 0.51% at Q=10 against 3.48% at Q=25), which is the mechanism above seen from the other side: more decisions per episode give more chances to select a member that triggers the repair.

### 5.6. The cb7 Diagnostic Under the Same Protocol

The diagnostic study of Section 4 was repeated under the protocol of this paper, so that the two benchmarks differ only in their instances: the thirty cb7 instances (n=100, m=30), five folds stratified by tightness, four chained training phases per fold, B=25,000, Q=25, twenty recorded seeds of their own, and 600 runs per strategy. Table 10 places the eleven strategies common to both campaigns side by side.

The table supports the saturation argument. On cb7, the eleven strategies span 0.11 percentage points of mean GAP, from 0.126% for SHADE to 0.237% for GA, against 7.6 points on cb9; PSO and Dolphin, more than an order of magnitude behind the leader on cb9, are within a factor of two of it on cb7; and the selection agent, second with 0.180%, is indistinguishable from round-robin (0.186%, p=0.77) and from uniform random selection (0.190%, p=0.42). The training records show the same from the inside: at the end of training the entropy of the policy stays above 99% of its maximum on every cb7 fold, whereas on cb9 it falls to between 94% and 97% as the policy learns to avoid PSO and Dolphin. Whether a selection problem exists is a property of the benchmark and the budget, and can be checked before training.

## 6. Discussion

The cross-validation results on cb9 answer RQ1 in part. The learned selection agent is ahead of round-robin and uniform random selection, the two strategies that select without looking at the search state, in four of the five folds and with $p<10^{-12}$ over the 600 paired scenarios; it is behind the strongest single method, Island GA, by 0.043 points and behind MGA. The co-evolution agent, which is the best learned policy on this benchmark, ends 0.015 points behind Island GA. The margins in both directions are negligible in effect size (${\hat{A}}_{12}$ between 0.45 and 0.55): on this benchmark eleven strategies lie within about 0.12 points of GAP, so the practical difference between recovering the best member and being told which one it is amounts to a few hundredths of a point. On cb9, the learned policies do not surpass a member that dominates the instance distribution, but come within 0.015 to 0.043 points of it without prior knowledge of which member that is while staying ahead of the blind alternatives.

The controls locate where the contribution of learning lies. The untrained agent, with the same architecture and environment, reaches a mean GAP 2.7 times higher with a standard deviation fourteen times larger, and the effect size against the trained agent is negligible: learning does not shift the typical run but removes the catastrophic ones, which in this portfolio come from selecting PSO or Dolphin, the two methods for which standalone GAP exceeds 6%. The trained policies select them in 2.0% and 7.1% of their decisions, almost entirely in one fold, and that fold is the agent’s worst. Consistent with this, retraining the agent on the portfolio without those two methods does not improve it (Section 5.3). The value of a learned selector over a portfolio depends on the portfolio containing members that must be avoided, and on the domain not having a member that dominates every instance. The repair-only baseline provides the lower bound: the operator alone accounts for a GAP of about 1%, and the portfolio and its orchestration for the reduction to below 0.4%.

The Set Covering Problem answers RQ2 and shows the other regime. With the state, the reward, the portfolio and the hyperparameters unchanged, and only the problem factory replaced, the selection agent ranks first of eleven strategies, has the best mean rank across folds and is the only strategy that leads more than one fold, while the leading single method changes from fold to fold and Island GA, which leads cb9, places fifth. The deterministic policies differ from fold to fold and concentrate on a few of the strong members without copying the leading single method of the fold; a selector that adapts its mixture to the fold is what a fixed choice cannot reproduce. Read together, the two domains delimit the operating conditions of the technique: the improvement signal must be informative at the budget used (the cb7 lesson: benchmark and budget jointly decide whether a selection problem exists, and the spread of the strategy ranking at the chosen budget is an inexpensive check for this), and the benefit over the best single method appears when no single member dominates. The budget study adds a third condition: at half the evaluation budget, the members that dominate cb9 lose their advantage on the loosest instances and the learned agents, together with any strategy that alternates methods, become the best options (Section 5.5). Read biomimetically, the cooperation of heterogeneous nature-inspired behaviors under a learned coordinator lets a portfolio approach or exceed the best single nature-inspired search strategy without knowing in advance which one it is.

The scope of these claims is set by the protocol: five folds, one training run per fold, twenty recorded seeds and 600 (cb9) or 1300 (SCP) paired scenarios per strategy. The dispersion of the fold means, between 0.03 and 0.05 points for the competitive strategies on cb9, combines the variation across instance subsets with that of the training run, and the two are not separated here. The joint selection-and-configuration agent remains behind the blind baselines at this training budget because one of its five policies collapses onto a weak configuration; the added configuration dimension needs a larger budget than the discrete selection it extends. The reward is shared by both domains without retuning, which the transfer to the SCP supports, but it is coarse: the first decision of an episode, which improves a random initial population, receives most of the reward mass, and a formulation that normalizes the improvement by the remaining gap is a natural refinement.

## 7. Conclusions

This article has presented a shared sequential-decision framework for orchestrating a portfolio of population-based metaheuristics with full population transfer, instantiated as three techniques of increasing action richness: discrete selection, joint selection and configuration through explicit translation maps from a shared configuration space to the native parameters of seven methods, and heterogeneous co-evolution through a simplex allocation over parallel demes. The evaluation uses five-fold stratified cross-validation with twenty recorded seeds, fifteen strategies including three controls, and a second binary domain, entered through the problem factory alone, on which the selection technique is evaluated against ten strategies.

On the cb9 instances at a budget where the improvement signal remains informative, the co-evolution agent reaches a mean GAP of 0.362% and the selection reaches agent 0.390%, against 0.417% and 0.418% for the two blind selectors, 1.042% for the untrained control, and 0.347% for Island GA. The latter is the strongest single metaheuristic, which neither agent surpasses. Relative to the better blind selector, the two agents reduce the mean GAP by 13% and 6.5%, and relative to the untrained control by 65% and 63%. At half the evaluation budget, the two agents rank first and second (0.396% and 0.427%), 11% and 4% below round-robin, while Island GA falls to 3.48%, almost nine times the GAP of the co-evolution agent. On 65 instances of the Set Covering Problem, where no single method leads every fold, the selection agent ranks first of eleven strategies with a mean GAP of 1.529%, 4.4% below the best single method (1.599%) and 27% below round-robin. It also has the best mean rank across folds, and is the only strategy that leads more than one fold. The controls show what the policy learns: an untrained network with the same architecture fails in a minority of runs by selecting the two portfolio members that collapse, and the trained policy avoids them; removing these members does not improve the trained agent (0.388% against 0.390%). The joint selection-and-configuration agent does not yet improve on the blind baselines at this budget. An earlier cb7 diagnostic established that the benchmark and budget jointly decide whether a selection problem exists. The shared formulation, translation maps, problem factory with two domains, and evaluation protocol with recorded seeds and controls are the reusable products of this study.

Beyond the specific results, the proposed framework may be viewed as a first step toward autonomous collective bio-inspired optimization systems in which heterogeneous biologically inspired search behaviors dynamically cooperate through learned decision mechanisms rather than fixed schedules. Casting a portfolio of nature-inspired metaheuristics as adaptive agents whose coordination is itself learned points to a broader research program on emergent self-organizing cooperation between biological search metaphors.

Future work motivated by these findings will add bandit and choice-function selection baselines and the best static configuration required by the dynamic algorithm configuration standard; scale the training of the joint selection-and-configuration agent; separate the training run variance from the instance variance with several training runs per fold; and revise the reward with a term normalized by the remaining gap, potential-based formulations of the shaping terms, and an autoregressive policy that conditions the configuration on the selected method. Extension to continuous domains, which requires a real-valued portfolio and state representation, is another direction. Further binary domains enter the framework through the same problem factory.

## Figures and Tables

**Table 1:** Related work compared with the present framework.

Work	Unit of Selection	Population	Learned Configuration	Decision Mechanism	Limitation for This Setting
Choice function [13]	low-level heuristics	one solution	no	online score	selects moves, not complete methods
Adaptive pursuit, bandit AOS [14,15]	operators of one evolutionary algorithm	one population, one algorithm	no	online credit	stateless beyond credit; one algorithm
DRL hyper-heuristic [16]	low-level heuristics	one solution	no	offline DRL	heuristics are moves, not metaheuristics
RL-based automated design [22]	algorithmic components of one search framework	one algorithm	no	offline RL	composes one algorithm; no selection among complete methods
DRL selection among DE variants [17]	complete DE variants	inherited between variants	no	offline DRL	one algorithm family; continuous problems
Dynamic algorithm configuration [18,19]	parameters of one algorithm	one algorithm	yes	offline RL (contextual MDP)	one algorithm; no selection among methods
AMALGAM [28]	complete metaheuristics, concurrent	shared (offspring shares)	no	fixed adaptation rule	rule-based, not learned; continuous problems
Population-based portfolios [29]	complete algorithms	separate, with migration	no	fixed budget division	no state-conditioned decision
Meta-hyper-heuristics [30]	complete metaheuristics	common	no	online adaptive selectors	rule-based; continuous problems
Selection HH on the MKP [36]	low-level heuristics, incl. crossover	one solution and a crossover memory	no	online selectors	operators; no learned policy over complete methods
This work	seven complete population-based metaheuristics	transferred in full at every decision	yes (translation maps) and simplex allocation	offline PPO over a 14-feature state	MKP cb9 and SCP under cross-validation

AOS: adaptive operator selection; DE: differential evolution; DRL: deep reinforcement learning; HH: hyper-heuristic; MDP: Markov decision process; MKP: multidimensional knapsack problem; PPO: proximal policy optimization; RL: reinforcement learning; SCP: set covering problem.

**Table 2:** Notation used in Section 3.

Symbol	Meaning	Value or Domain
**Problems**		
*n*, *m*	number of items (columns) and of resources (rows)	instance
$p_{j}$, $c_{i}$, $r_{ij}$	MKP profits, capacities and consumptions	instance
$w_{j}$, $a_{ij}$	SCP column costs and covering matrix	instance
*x*, f(x)	binary solution and its objective value	$x\in {\left\{0,1\right\}}^{n}$
$f^{\mathrm{ref}}$, GAP	reference value of the instance; relative gap to it (%)	Section 3.1
**Decision process**		
M=(S,A,P,R,γ)	Markov decision process of the hyper-heuristic	
*k*	number of portfolio metaheuristics	7
*B*, *Q*, *N*	evaluation budget, quantum (iterations per decision), population size	25,000; 25; 100
*t*, $e_{t}$	decision index; evaluations consumed up to *t*	$e_{t}\leq B$
$P_{t}$, ${\mathrm{MH}}_{a}$	shared population at decision *t*; metaheuristic with index *a* applied to it	
$x^{*}$, $f_{t}^{*}$	incumbent and its value after decision *t*	
$s_{t}$, *d*	state vector and its dimension	$s_{t}\in {\left[0,1\right]}^{d}$, d=14
$a_{t}$, $\pi_{\theta }$	selected metaheuristic; policy with parameters θ	$a_{t}\in \left\{0,\ldots ,k-1\right\}$
**State features**		
$s_{t}^{\mathrm{ISM}}$, ${\mathrm{ISM}}_{t}$	stagnation signal; iterations since the last improvement	${\mathrm{ISM}}_{\max}=200$
$D_{t}$, $q_{j}$	mean per-gene binary entropy (diversity); frequency of gene *j*	$D_{t}\in \left[0,1\right]$
$H_{t}$	mean normalized Hamming distance to the incumbent	[0,1]
$s_{t}^{\mathrm{col}}$	diversity-collapse signal	${\mathrm{col}}_{\max}=100$
$\iota_{t}$, $\nu_{t}$, $\tau_{t}$	recent-improvement indicator, offspring acceptance rate, budget fraction consumed	[0,1]
$e(a_{t-1})$	one-hot encoding of the previous action	${\left\{0,1\right\}}^{k}$
**Joint selection and configuration**		
$\tilde{a}=\left(a,\kappa \right)$	hybrid action: method index and configuration vector	$\kappa \in {\left[0,1\right]}^{q}$, q=3
$\kappa_{e}$, $\kappa_{x}$, $\kappa_{p}$	exploration intensity, exploitation intensity, population pressure	eleven levels each
$\varphi_{a}$, $\Theta_{a}$, $\theta_{a}^{\mathrm{def}}$	translation map of method *a*, its native parameter space and default configuration	Appendix A
$\pi_{\theta }^{\mathrm{sel}}$, $\pi_{\theta }^{\mathrm{cfg}}$	selection head and configuration head of the policy	
**Heterogeneous co-evolution**		
$z_{t}$, $\alpha_{t}$	continuous action and its softmax image on the simplex	$z_{t}\in {\left[-10,10\right]}^{k}$, $\alpha_{t}\in \Delta^{k-1}$
η, $K_{t}$, $N_{t,a}$	activation floor, active set, deme size of method *a*	η=0.10
μ, $d_{C}$	fusion operator of the demes; dimension of the extended state	$d_{C}=28$
**Reward and learning**		
$r_{t}$; $r_{t}^{I}$, $r_{t}^{S}$, $r_{t}^{D}$	reward; improvement, stagnation and diversity-recovery terms	Section 3.7
$w_{I}$, $w_{S}$, $w_{D}$, $\lambda_{S}$	reward weights and stagnation coefficient	1.0, 0.3, 0.2, 0.5
ε	tolerance below which the relative improvement is not computed	
$V_{\phi }$, γ, λ, ϵ	value network; discount, GAE parameter and clip range of PPO	0.99, 0.95, 0.2
*E*, *M*, $\theta^{*}$	PPO epochs and minibatch size; optimal policy parameters	Algorithm 4
**Evaluation**		
α	tightness ratio of an MKP instance	0.25, 0.50, 0.75
${\hat{A}}_{12}$, $\sigma_{\mathrm{folds}}$	Vargha–Delaney effect size; standard deviation of the fold means	Section 4.2

Values are those of the reference configuration; the reward weights and PPO hyperparameters are given in Section 3.7 and Section 3.8.

**Table 3:** Portfolio of low-level metaheuristics.

*a*	Metaheuristic	Family	Search Mechanism
0	GA [2]	Evolutionary	Tournament selection, crossover, adaptive mutation
1	PSO [8]	Swarm	Velocity with inertia, cognitive and social terms
2	EDA [6]	Probabilistic model	Univariate marginal model (UMDA) and sampling
3	SHADE [7]	Differential evolution	Current-to-pbest mutation, history-based F,CR
4	MGA [4]	Memetic	GA with first-improvement local search
5	Dolphin [9,48]	Swarm	Probabilistic model updated by echolocation
6	Island GA [5]	Evolutionary (parallel)	Multi-population with ring migration

*F* and CR: scale factor and crossover rate of differential evolution; UMDA: univariate marginal distribution algorithm.

**Table 4:** Ranking of the fifteen strategies on cb9 under five-fold cross-validation.

#	Strategy	GAP (%)	SD	${\hat{A}}_{12}$	Magnitude	Wilcoxon *p*
1	Island GA alone	0.3470	0.2093	0.445	negligible	<$10^{-4}$
2	PPO agent (co-evolution)	0.3624	0.2196	0.466	negligible	<$10^{-4}$
3	MGA alone	0.3706	0.2144	0.487	negligible	<$10^{-4}$
4	PPO agent (5-MH portfolio) ^†^	0.3879	0.2431	0.490	negligible	0.307
5	**PPO agent (selection)**	0.3895	0.2369	—	—	—
6	GA alone	0.4053	0.2436	0.524	negligible	<$10^{-4}$
7	Round-robin selection	0.4166	0.2422	0.547	negligible	<$10^{-4}$
8	Uniform random selection	0.4183	0.2413	0.546	negligible	<$10^{-4}$
9	SHADE alone	0.4318	0.2609	0.551	negligible	<$10^{-4}$
10	PPO agent (selection + configuration)	0.4605	0.3731	0.529	negligible	<$10^{-4}$
11	EDA alone	0.4673	0.2731	0.596	small	<$10^{-4}$
12	Repair-only baseline ^†^	1.0073	0.7388	0.767	large	<$10^{-4}$
13	Untrained PPO agent ^†^	1.0421	3.3535	0.548	negligible	<$10^{-4}$
14	Dolphin alone	6.4243	8.0697	0.881	large	<$10^{-4}$
15	PSO alone	7.9078	9.7726	0.939	large	<$10^{-4}$

GAP: mean over the runs; SD: standard deviation over the runs. Every instance is evaluated by the model of the fold that never saw it during training, with the twenty recorded evaluation seeds: 30×20=600 runs per strategy, B=25,000, complete repair operator. GAP against the best-known values of the literature [11,12]. ${\hat{A}}_{12}$ and the two-sided paired Wilcoxon *p*-value compare the selection agent against each strategy; ${\hat{A}}_{12}>0.5$ means the selection agent attains the lower GAP, with magnitudes following the Vargha–Delaney thresholds [53] recommended by Arcuri and Briand [54]. ^†^ Control strategies: the untrained agent isolates the contribution of learning from that of the architecture and the environment, the five-metaheuristic agent is the portfolio ablation without PSO and Dolphin, and the repair-only baseline measures the repair operator on its own. Bold: the selection agent, the reference strategy of the comparison.

**Table 5:** Mean GAP (%) per fold on cb9.

Strategy	Fold 0	Fold 1	Fold 2	Fold 3	Fold 4	Mean	$\sigma_{\mathrm{folds}}$
Island GA alone	0.4017	0.3240	0.3311	0.3282	0.3497	0.3470	0.0322
PPO agent (co-evolution)	0.4167	0.3275	0.3379	0.3273	0.4028	0.3624	0.0437
MGA alone	0.4153	0.3365	0.3613	0.3509	0.3889	0.3706	0.0315
PPO agent (5-MH portfolio) ^†^	0.4678	0.3449	0.3572	0.3627	0.4068	0.3879	0.0504
**PPO agent (selection)**	0.3977	0.3819	0.3525	0.4541	0.3613	0.3895	0.0402
GA alone	0.4648	0.3690	0.3915	0.3826	0.4186	0.4053	0.0379
Round-robin selection	0.4704	0.3854	0.3952	0.3887	0.4435	0.4166	0.0381
Uniform random selection	0.4672	0.3858	0.4122	0.3927	0.4338	0.4183	0.0331
SHADE alone	0.4957	0.3978	0.4182	0.4006	0.4468	0.4318	0.0407
PPO agent (selection + configuration)	0.7120	0.3736	0.4177	0.4019	0.3972	0.4605	0.1415
EDA alone	0.5391	0.4276	0.4458	0.4350	0.4892	0.4673	0.0467
Repair-only baseline ^†^	1.1152	0.9430	1.0320	0.9252	1.0211	1.0073	0.0764
Untrained PPO agent ^†^	1.2025	0.9997	1.0765	0.8641	1.0677	1.0421	0.1235
Dolphin alone	6.4768	6.4443	6.4251	6.3125	6.4628	6.4243	0.0655
PSO alone	7.9310	7.9418	7.8570	7.8398	7.9692	7.9078	0.0563

Six held-out instances per fold and twenty evaluation seeds (120 runs per cell). $\sigma_{\mathrm{folds}}$ is the standard deviation of the five fold means: how much each strategy depends on the split of instances and, for the learned agents, on the training run. Bold: the selection agent, the reference strategy of the comparison. ^†^ Control strategies, described in the footnote of Table 4.

**Table 6:** Outcome of the training decisions of the five cb9 agents per selected method.

Method	Improved (%)	After 5th (%)	P95 Gain (%)	Share at Eval. (%)
MGA	64.4	45.0	10.9	9.0
Island GA	61.9	44.0	7.9	30.1
GA	56.0	35.1	9.4	0.5
SHADE	43.1	21.0	14.8	27.9
EDA	39.7	17.4	14.9	23.5
Dolphin	10.6	0.08	1.15	7.1
PSO	5.5	0.05	0.08	2.0

95,372 decisions, first decision of each episode excluded. Improved: share of decisions that improved the best solution of the episode; After 5th: the same share restricted to decisions after the fifth of an episode; P95 gain: 95th percentile of the relative improvement per decision; Share at eval.: share of each method in the decisions of the deterministic policies at evaluation (Section 5.1).

**Table 7:** Ranking of the eleven strategies on the Set Covering Problem under five-fold cross-validation.

#	Strategy	GAP (%)	SD	${\hat{A}}_{12}$	Magnitude	Wilcoxon *p*
1	**PPO agent (selection)**	1.5293	2.0434	—	—	—
2	GA alone	1.5994	2.0058	0.522	negligible	<$10^{-3}$
3	MGA alone	1.6261	1.9989	0.527	negligible	<$10^{-4}$
4	SHADE alone	1.6792	2.4006	0.480	negligible	0.012
5	Island GA alone	1.7021	2.0201	0.541	negligible	<$10^{-4}$
6	Untrained PPO agent ^†^	1.9032	2.2064	0.559	negligible	<$10^{-4}$
7	Round-robin selection	2.1056	2.3536	0.579	small	<$10^{-4}$
8	Uniform random selection	2.3364	2.3977	0.610	small	<$10^{-4}$
9	EDA alone	2.5825	2.5357	0.642	medium	<$10^{-4}$
10	Dolphin alone	3.3805	2.3241	0.754	large	<$10^{-4}$
11	PSO alone	3.4794	2.3611	0.764	large	<$10^{-4}$

Same protocol and same twenty recorded seeds as Table 4: 65 × 20 = 1300 runs per strategy. State, reward and portfolio reused without change; the domain enters through the problem factory. GAP against the optimal or best-known values of the OR-Library. Columns as in Table 4. Bold: the selection agent, the reference strategy of the comparison. ^†^ Control strategies, described in the footnote of Table 4.

**Table 8:** Mean GAP (%) per fold on the Set Covering Problem.

Strategy	Fold 0	Fold 1	Fold 2	Fold 3	Fold 4	Mean	$\sigma_{\mathrm{folds}}$
**PPO agent (selection)**	0.8321	1.0432	2.3986	2.2837	1.0888	1.5293	0.7485
GA alone	0.9307	1.2666	2.5555	2.2356	1.0087	1.5994	0.7459
MGA alone	0.9107	1.3939	2.5126	2.1802	1.1329	1.6261	0.6895
SHADE alone	0.8364	0.8616	2.9302	2.6128	1.1552	1.6792	1.0112
Island GA alone	1.0982	1.4289	2.5686	2.2459	1.1690	1.7021	0.6652
Untrained PPO agent ^†^	1.2229	1.4661	2.8437	2.6616	1.3220	1.9032	0.7828
Round-robin selection	1.4011	1.5971	3.0642	2.9837	1.4822	2.1056	0.8416
Uniform random selection	1.7050	1.8579	3.2492	3.1530	1.7168	2.3364	0.7924
EDA alone	1.9521	1.7624	3.7405	3.6230	1.8346	2.5825	1.0066
Dolphin alone	2.8119	2.9883	4.5281	3.9625	2.6119	3.3805	0.8251
PSO alone	2.8967	3.0480	4.6329	4.0540	2.7655	3.4794	0.8210

Thirteen held-out instances per fold and twenty evaluation seeds. No single metaheuristic leads every fold: SHADE leads fold 1, MGA fold 3 and GA fold 4, while the selection agent leads folds 0 and 2 and has the best mean rank across folds (2.0, against 2.6 for GA and MGA, the only other strategies that stay in the top four). Bold: the selection agent, the reference strategy of the comparison. ^†^ Control strategies, described in the footnote of Table 4.

**Table 9:** Effect of halving the evaluation budget on cb9.

Strategy	*B* = 25,000		*B* = 12,500		Δ
	GAP (%)	Rank	GAP (%)	Rank	
PPO agent (co-evolution)	0.362	2	0.396	1	+0.03
**PPO agent (selection)**	0.390	4	0.427	2	+0.04
Round-robin selection	0.417	6	0.447	3	+0.03
EDA alone	0.467	10	0.492	4	+0.02
SHADE alone	0.432	8	0.500	5	+0.07
PPO agent (selection + configuration)	0.460	9	0.569	6	+0.11
Uniform random selection	0.418	7	1.103	7	+0.68
Untrained PPO agent	1.042	11	3.177	8	+2.13
Island GA alone	0.347	1	3.480	9	+3.13
GA alone	0.405	5	3.639	10	+3.23
MGA alone	0.371	3	5.294	11	+4.92
Dolphin alone	6.424	12	6.578	12	+0.15
PSO alone	7.908	13	8.134	13	+0.23

The 600 scenarios, the twenty recorded seeds and the trained models of Table 4 re-evaluated with B=12,500; the rank is among the thirteen strategies of this comparison. Δ: mean paired increase in GAP; every increase is significant (two-sided paired Wilcoxon signed-rank test, $p<10^{-4}$). Bold: the selection agent, the reference strategy of the comparison.

**Table 10:** The eleven common strategies on cb7 and cb9 under the same protocol.

Strategy	cb7 (*n* = 100)			cb9 (*n* = 500)		
	GAP (%)	SD	*p*	GAP (%)	SD	*p*
SHADE alone	0.126	0.164	<10^−4^	0.432	0.261	<10^−4^
**PPO agent (selection)**	0.180	0.189	—	0.390	0.237	—
Round-robin selection	0.186	0.205	0.768	0.417	0.242	<10^−4^
Uniform random selection	0.190	0.219	0.417	0.418	0.241	<10^−4^
MGA alone	0.204	0.227	0.010	0.371	0.214	<10^−4^
Untrained PPO agent	0.205	0.216	0.002	1.042	3.354	<10^−4^
Dolphin alone	0.212	0.206	<10^−4^	6.424	8.070	<10^−4^
PSO alone	0.212	0.191	<10^−4^	7.908	9.773	<10^−4^
EDA alone	0.223	0.242	<10^−4^	0.467	0.273	<10^−4^
Island GA alone	0.223	0.245	<10^−4^	0.347	0.209	<10^−4^
GA alone	0.237	0.259	<10^−4^	0.405	0.244	<10^−4^

Rows ordered by the cb7 GAP. Same protocol on both sets: five folds stratified by tightness, four chained training phases per fold, twenty recorded seeds, 600 runs per strategy, B=25,000, Q=25. *p*: two-sided paired Wilcoxon signed-rank test against the selection agent. The cb9 values are those of Table 4. Bold: the selection agent, the reference strategy of the comparison.

## Data Availability

The data supporting this article, including the thirty cb9, the thirty cb7 and 65 Set Covering Problem instances with provenance, the reference values (Appendix B), the fold compositions and the registry of the twenty evaluation seeds, the raw per-run results of every strategy in both domains, the training logs and trained models of each fold, the analysis scripts and the complete source code of the framework (environment, the three action-space formulations, translation maps, portfolio, training, evaluation and cross-validation engines), are openly available in the public repository https://github.com/cacc92/drl-metaheuristic (accessed on 9 September 2026), as the nine archives described in its README. The benchmark instances are those distributed by the OR-Library. The cb9 and cb7 sets are the files https://people.brunel.ac.uk/~mastjjb/jeb/orlib/files/mknapcb9.txt (accessed on 9 September 2026) and https://people.brunel.ac.uk/~mastjjb/jeb/orlib/files/mknapcb7.txt (accessed on 9 September 2026), with the format described at https://people.brunel.ac.uk/~mastjjb/jeb/orlib/mknapinfo.html (accessed on 9 September 2026). The 65 Set Covering instances are the files scp41–scp410, scp51–scp510, scp61–scp65, scpa1–scpa5, scpb1–scpb5, scpc1–scpc5, scpd1–scpd5, scpnre1–scpnre5, scpnrf1–scpnrf5, scpnrg1–scpnrg5 and scpnrh1–scpnrh5 of the same directory (https://people.brunel.ac.uk/~mastjjb/jeb/orlib/files/scp41.txt (accessed on 9 September 2026) and so on), with the format described at https://people.brunel.ac.uk/~mastjjb/jeb/orlib/scpinfo.html (accessed on 9 September 2026). The transcription of the instances and the OR-Library values of 1998 for cb9 can be verified from the official files included in the repository; the reference values taken from the literature are listed with their sources in the repository.

## Appendix A. Translation Maps

The seven translation maps from the shared configuration space $\kappa =(\kappa_{e},\kappa_{x},\kappa_{p})$ to native parameters are:

- GA: $p_{m}=0.05+0.45\;\kappa_{e}$; $p_{c}=0.60+0.35\;\kappa_{x}$; $t_{s}=\left\lceil 2+3\;\kappa_{p}\right\rceil$.
- PSO: $\omega =0.40+0.50\;\kappa_{e}$; $c_{1}=1.00+1.20\;\kappa_{x}$; $c_{2}=1.00+1.20\;\kappa_{p}$.
- EDA: $p_{f}=0.01+0.09\;\kappa_{e}$; $\eta =0.30+0.70\;\kappa_{x}$; $s_{r}=0.70-0.50\;\kappa_{p}$.
- SHADE: $F_{0}=0.30+0.50\;\kappa_{e}$; $CR_{0}=0.30+0.60\;\kappa_{x}$; $p_{b}=0.25-0.20\;\kappa_{p}$.
- MGA: $p_{m}=0.05+0.45\;\kappa_{e}$; $l_{f}=\left\lceil 10-9\;\kappa_{x}\right\rceil$; $l_{m}=\left\lfloor 5+45\;\kappa_{p}\right\rfloor$.
- Dolphin echolocation: $pp_{1}=0.05+0.25\;\kappa_{e}$; $\beta =0.50+1.50\;\kappa_{x}$ (two coordinates; $\kappa_{p}$ unused).
- Island GA: $m_{i}=\left\lceil 3+17\;\kappa_{e}\right\rceil$; $m_{s}=\left\lceil 1+4\;\kappa_{x}\right\rceil$; $p_{m}=0.05+0.45\;\kappa_{p}$.

Default levels reproduce the literature settings of each method, so the pure-selection formulation is recovered at $\kappa =\kappa^{\mathrm{def}}$.

## Appendix B. Reference Values of the cb9 Instances

For each of the thirty cb9 instances, Table A1 lists the value distributed with the OR-Library results file obtained by the genetic algorithm of Chu and Beasley [32], the best-known value reported by Lai et al. [11], and the value used as reference in this study, which incorporates the three improvements published by Xu et al. [12]. All GAP figures in the paper are computed against the last numerical column.

**Table A1:** Reference values of the cb9 instances.

OR-Library id	α	OR-Library (1998)	Best-Known 2018	Reference Used	Source
30.500-00	0.25	115,868	116,056	116,056	[11]
30.500-01	0.25	114,667	114,810	114,810	[11]
30.500-02	0.25	116,661	116,741	116,741	[11]
30.500-03	0.25	115,237	115,354	**115,370 **	[12]
30.500-04	0.25	116,353	116,525	**116,539**	[12]
30.500-05	0.25	115,604	115,741	115,741	[11]
30.500-06	0.25	113,952	114,181	114,181	[11]
30.500-07	0.25	114,199	114,348	114,348	[11]
30.500-08	0.25	115,247	115,419	115,419	[11]
30.500-09	0.25	116,947	117,116	117,116	[11]
30.500-10	0.50	217,995	218,104	218,104	[11]
30.500-11	0.50	214,534	214,648	214,648	[11]
30.500-12	0.50	215,854	215,978	215,978	[11]
30.500-13	0.50	217,836	217,910	217,910	[11]
30.500-14	0.50	215,566	215,689	215,689	[11]
30.500-15	0.50	215,762	215,919	215,919	[11]
30.500-16	0.50	215,772	215,907	215,907	[11]
30.500-17	0.50	216,336	216,542	216,542	[11]
30.500-18	0.50	217,290	217,340	**217,364**	[12]
30.500-19	0.50	214,624	214,739	214,739	[11]
30.500-20	0.75	301,627	301,675	301,675	[11]
30.500-21	0.75	299,985	300,055	300,055	[11]
30.500-22	0.75	304,995	305,087	305,087	[11]
30.500-23	0.75	301,935	302,032	302,032	[11]
30.500-24	0.75	304,404	304,462	304,462	[11]
30.500-25	0.75	296,894	297,012	297,012	[11]
30.500-26	0.75	303,233	303,364	303,364	[11]
30.500-27	0.75	306,944	307,007	307,007	[11]
30.500-28	0.75	303,057	303,199	303,199	[11]
30.500-29	0.75	300,460	300,596	300,596	[11]

OR-Library value, best-known value of 2018, and reference used in this study. Bold marks the three values improved after 2018.
